# Mechanical properties of fibre-reinforced geopolymer-cemented tailings used as backfill

**DOI:** 10.1371/journal.pone.0314617

**Published:** 2024-12-05

**Authors:** Xueqiang Sha, Chao Cheng, Guoyong Pan, Zitao Zhu, Chunxiao Qi, Weidong Chen

**Affiliations:** China Construction Sixth Bureau Civil Engineering Co.Ltd., Tianjin, China; SASTRA Deemed University, INDIA

## Abstract

Backfill materials are used in underground engineering to fill voids and buried excavated parts. In this study, solid waste was utilised as a raw material mixed with different amounts of polypropylene fibres to determine the optimal sodium hydroxide content, water—solid ratio, and fibre content. The uniaxial compressive strength (UCS) of the produced backfill materials was measured, and the interfacial structures were analysed via scanning electron microscopy. The results revealed that the mechanical properties of the backfill materials were influenced in the order sodium hydroxide doping > water—solid ratio > fibre doping. The optimal material composition corresponded to a sodium hydroxide content of 3%, water—solid ratio of 0.28, and fibre content of 5 ‰. The slag produced a C—S–H gel. Meanwhile, the fly ash and gangue contained large amounts of aluminium, which formed hydrated aluminosilicates. The addition of polypropylene fibres reduced the number of internal defects in the backfill structure and increased the UCS.

## 1. Introduction

Iron tailings sand is discharged worldwide as beneficiation waste at a rate of more than 10 billion tons per year; however, the utilisation rate of tailings in China is only 7%. Therefore, the comprehensive recycling of iron tailing sand is a highly important task [1, 2]. Blast furnace slag is a type of solid waste, and its large stockpiling amount is hazardous to the environment, and represents a waste of resources [3]. Fly ash is a volcanic ash material. When this material is discharged into a water system, it causes the siltation of rivers, and the toxic chemicals present in sly ash may harm human beings and other living organisms [4, 5].

The comprehensive utilisation of iron tailing sand worldwide has received significant attention from researchers. For example, it has been widely used as a concrete filler [6], road base material [7–9], and filling material [10–12]. Sun et al. [13, 14] prepared a fly ash geopolymer gangue aggregate filling body by using fly ash as a cementitious material and established a deformation localisation law of the filling body via a digital scattering correlation method. Wang et al. [15] used limestone tailings sand to prepare a limestone tailings-based cementing material and improved its performance by optimising the pore structure. Qiu et al. [16] used the emerging microbial‑induced carbonate precipitation technology to promote iron tailing consolidation and determined the strength difference between a blank group and the microbially cemented iron tailing-based backfill. Zhang et al. [17] tested the activity of ultrafine tailings by using ultrafine tailings to prepare clinker-free backfill and exploring its filling performance and hydration mechanism. Although multiple studies on alkali-excited cementitious materials have been conducted, such materials have rarely been applied in practice because factors affecting their performance have not been investigated systematically. Freeze—thaw damage is a destructive process that occurs periodically owing to the freezing and expansion forces, which change the strength and other mechanical properties of a rock mass. Li et al. [18] analysed the influence of geometrical characteristics of fissures on the strength of rock bodies by performing freeze—thaw cycle and uniaxial compression experiments. Moreover, the authors established a deterioration model for the freeze—thaw loaded fissured rock based on the microscopic damage and macroscopic statistical theories to elucidate the damage deterioration mechanism of the fissured rock body under the coupling effect of freezing, thawing, and loading. Fu et al. [19] prepared high-performance alkali slag concrete (ASC) using a composite slag exciter, Na_2_SiO_3_, and NaOH and investigated its freeze—thaw durability, microstructure, performance, and variation rules of internal damage variables after freezing and thawing. They also modelled ASC damage by conducting freeze—thaw cycling tests, scanning electron microscopy (SEM) observations, and energy spectrum analyses. Chen et al. [20] performed road performance and freeze—thaw cycle tests on pulverised clay and its cured soil using alkali-excited materials as the curing agents. The microscopic characteristics of the cured soil were investigated via SEM and X-ray diffraction (XRD), and the effect of the alkali-excited materials on the road performance index and freeze—thaw resistance characteristics of the pulverised clay were explored.

Numerous studies have been conducted on the static and kinetic properties of alkali-excited materials [21–27]. In this study, the sodium hydroxide doping amount, water—solid ratio, and polypropylene fibre content of polypropylene fibre-reinforced backfill materials were optimised using an orthogonal design method to obtain a cementitious material suitable for landfill excavation. Its mechanical properties and microstructure were correlated with the backfill internal structure and strength enhancement mechanism.

## 2. Materials and testing procedure

### 2.1 Test materials

**Fig 1:**
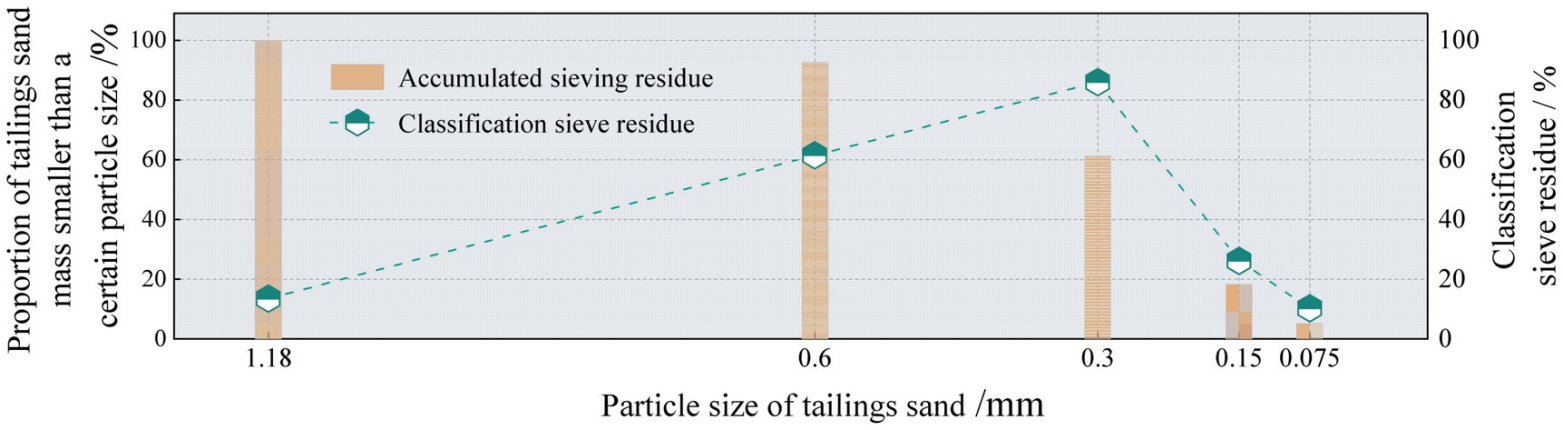
Particle grading curve of the iron tailing sand.

**Fig 2:**
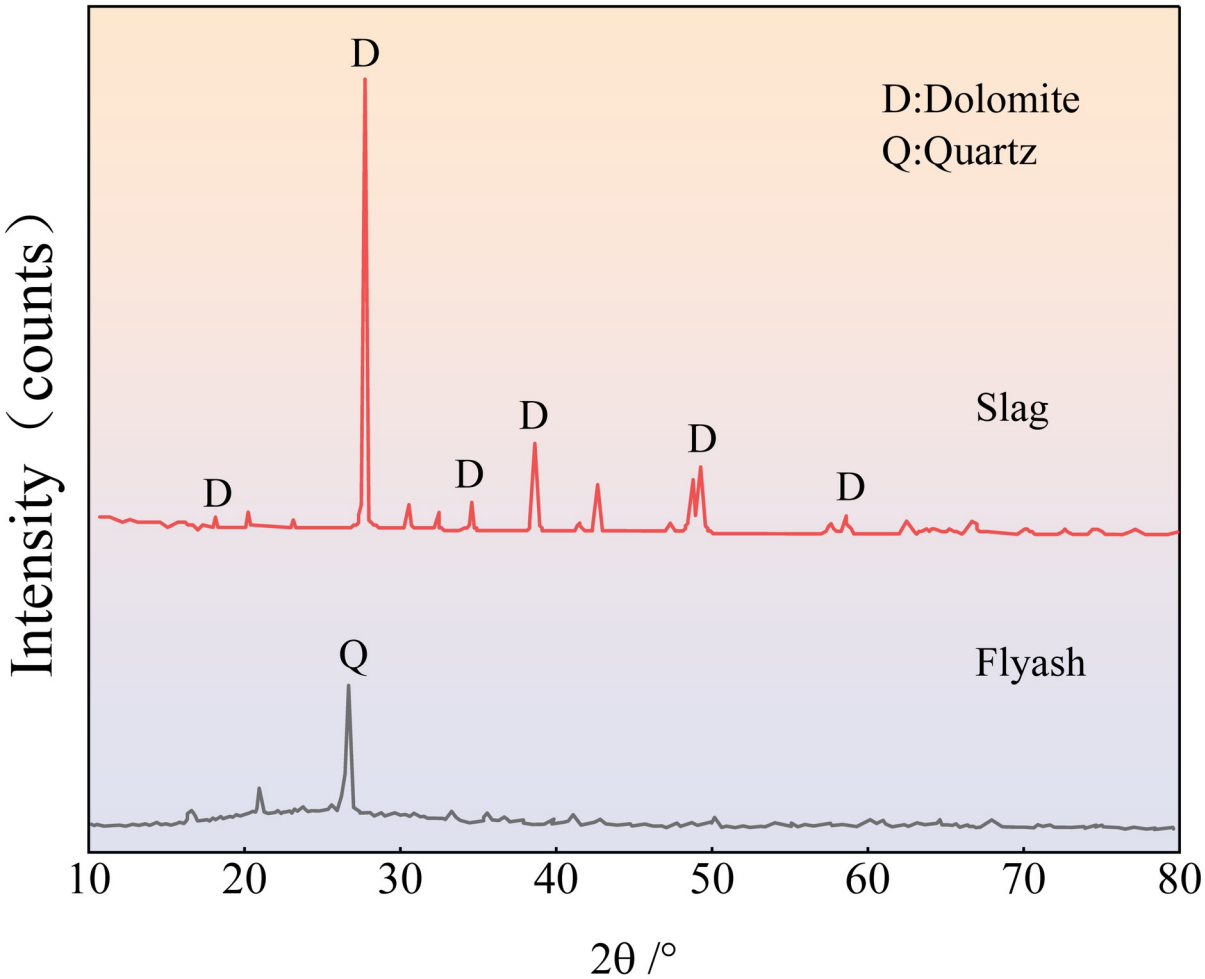
XRD patterns of the slag and fly ash.

**Fig 3:**
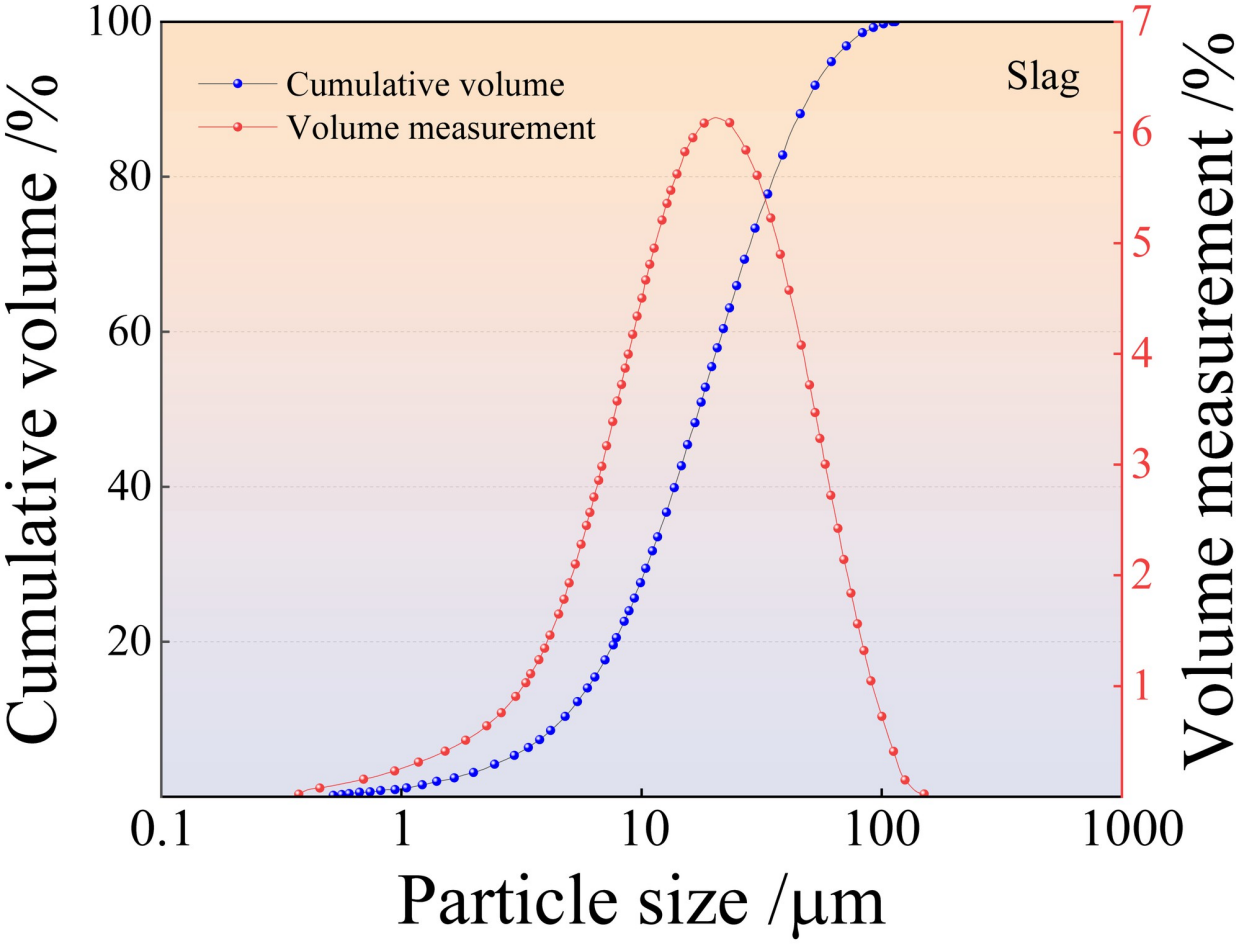
Particle size distributions of the slag.

**Fig 4:**
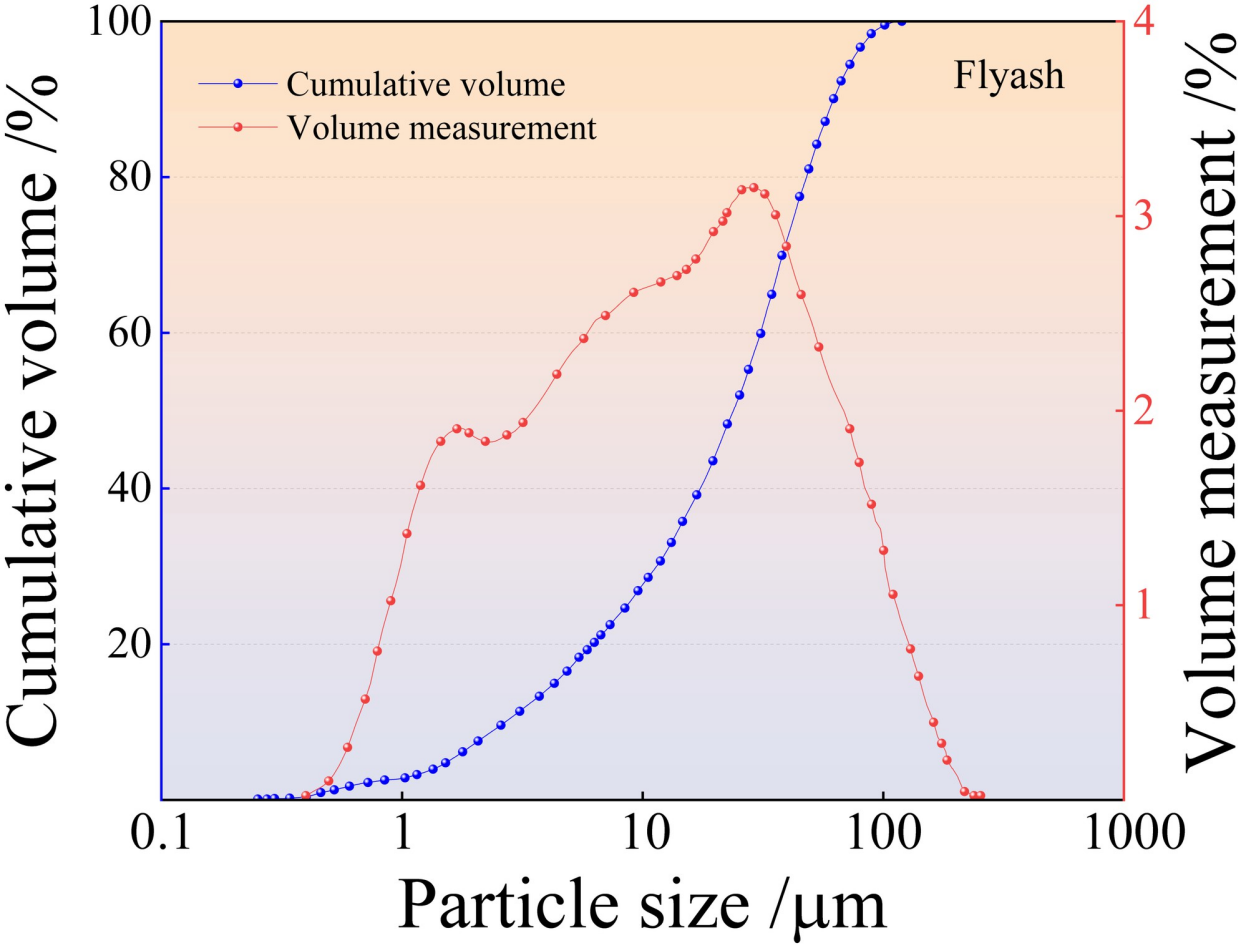
Particle size distributions of fly ash.

**Fig 5:**
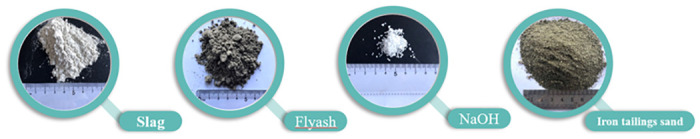
Photographs of the raw materials.

The solid raw materials used in this study were tailings, fly ash, and slag. Fuxin tap water was used for testing, and the alkaline exciter was composed of sodium hydroxide granules. Iron tailings sand was obtained from the Fengshuigou tailing pond of the Qidashan ore dressing plant of the Angang Mining Group. Its physical properties are listed in Table 1, and the particle grading curve is shown in Fig 1. Fly ash was obtained from the Rizhao Huaneng Power Plant, and slag was supplied by Henan Yuanheng Environmental Protection Engineering Company Limited. Their chemical compositions were determined using X-ray fluorescence spectrometry. The results are listed in Table 2. The physical properties of the slag are summarised in Table 3. The phase structures of the slag and fly ash were obtained using Rigaku Benchtop XRD (MiniFlex 600) instrument. The resulting diffraction patterns are shown in Fig 2. The particle size distributions of the slag and fly ash were determined using a Bettersize-2000 laser particle size analyser. The curves obtained are shown in Figs 3 and 4. Polypropylene fibres were produced by Shandong Yishun Engineering Materials Co., and their corresponding physical properties are listed in Table 4. Photographs of the raw materials used in this study are shown in Fig 5.

**Table 1:** Physical properties of the iron tailings sand.

Gravity/kN·m^-3^	Modulus of deformation /MPa	Poisson’s ratio	Cohesive strength /kPa	Angle of internal friction /°	Unevenness coefficient	Curvature coefficient
17.41	30.02	0.26	1.01	33.43	3.04	1.21

**Table 2:** Chemical compositions of the fly ash and slag (wt/%).

Chemical composition	CaO	Fe_2_O_3_	SiO_2_	Al_2_O_3_	MgO	Na_2_O	K_2_O	SO_3_	FeO	S
Fly ash	4.14	3.76	58.77	25.14	0.46	0.97	1.18	0.37	–	–
Slag	44.35	0.37	33.26	13.26	6.33	0.32	0.27	–	0.89	1.1

**Table 3:** Physical properties of the slag.

Density / g·cm^−3^	Specific surface area /m^2^·kg^−1^	Burning vector /%	Chlorion /%	Mobility ratio /%	Water content /%
2.9	428	0.21	0.024	109	0.32

**Table 4:** Physical properties of polypropylene fibres.

Type	Length /mm	Density/ g·cm^−3^	Tensile strength /MPa	Elastic modulus /GPa	Melting point /°C	Ignition point /°C	Fracture elongation /%	Dispersibility
Polypropylene fibres	12	0.91	≥300	≥3.5	165	590	≥15	Fabulous

### 2.2 Testing procedure

A three-factor three-level orthogonal test (L9(34)) was designed to investigate the effects of NaOH dosing (A), water—solid ratio (B), and fibre dosing (C) on the compressive properties of the geopolymer-gelatinised cemented end-sand specimens. The values of each factor are listed in Table 5.

**Table 5:** Orthogonal experiment design factors and levels.

Level	Factor		
	A	B	C
1	1	0.24	4
2	2	0.28	5
3	3	0.32	6

**Fig 6:**
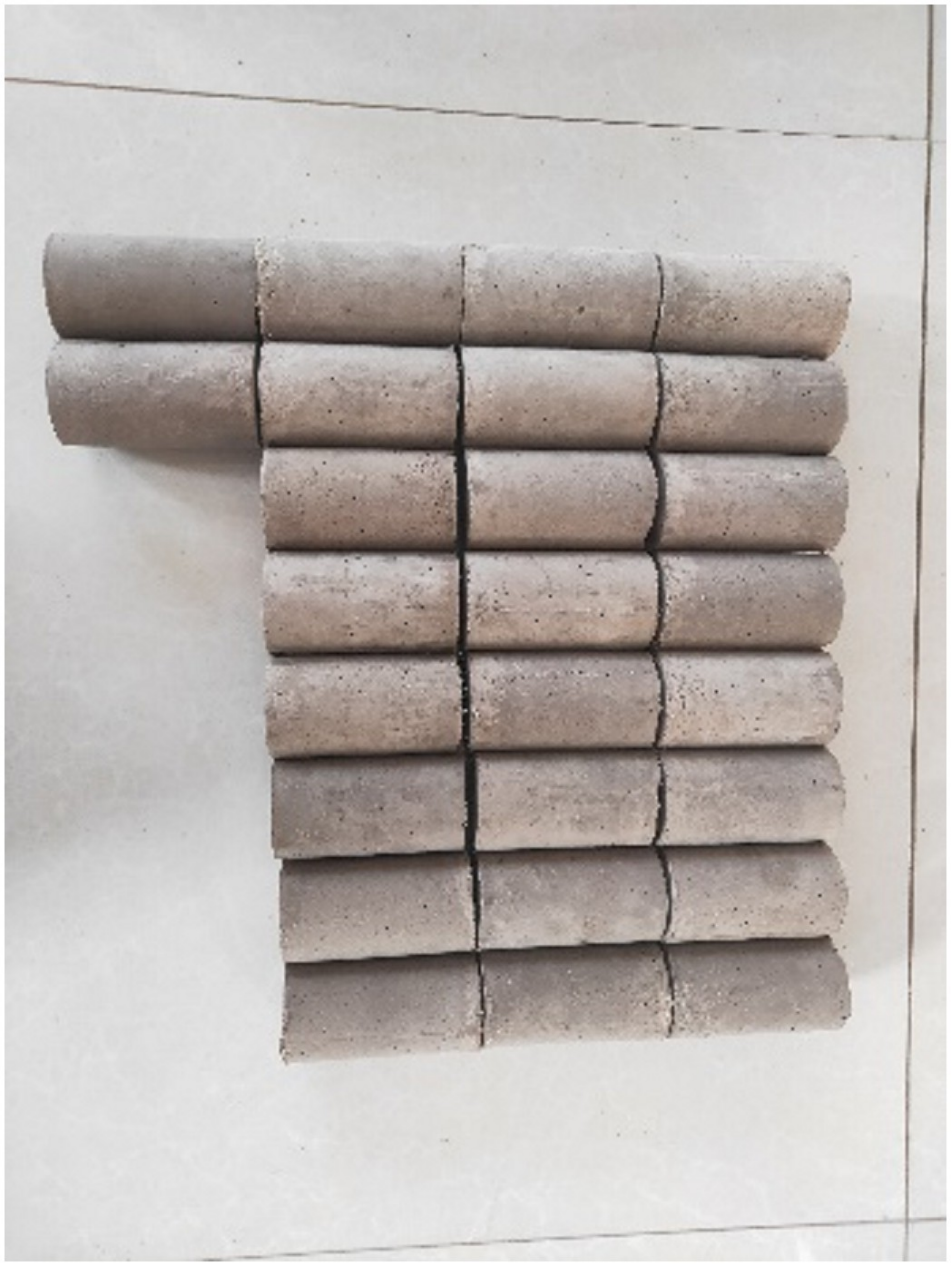
Partial specimen.

Specimens were prepared according to the test design quality ratio. Appropriate amounts of the raw materials were weighed, placed in a glue sand mixer, and mixed at a uniform speed for 3 min. Water and sodium hydroxide were mixed at an appropriate ratio, cooled to room temperature, and poured into the glue sand mixer. The resulting system was stirred quickly for 3–5 min until the slurry achieved a certain degree of mobility and then uniformly poured into a film tool (φ 50 mm × 100 mm) and vibration-compacted on a vibration table for 24 h. Demoulding was performed in a standard maintenance box after 3, 7, and 28 d of aging. Three parallel specimens were produced for each ratio (Fig 6).

**Fig 7:**
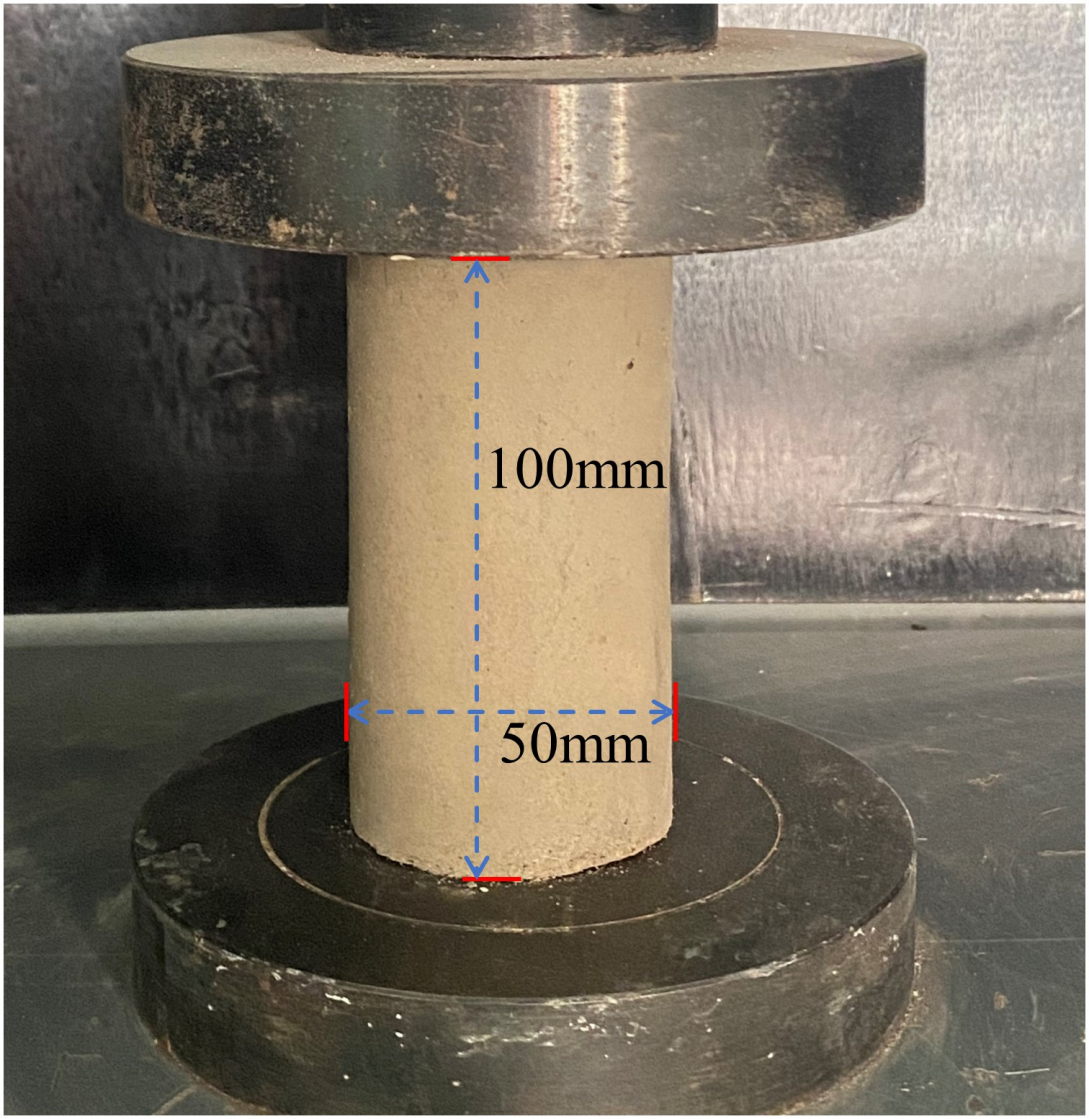
Loading process.

During testing, a WDW-100E testing machine was used to perform uniaxial compression tests on the specimens that satisfied the testing requirements. The loading process illustrated in Fig 7 was conducted in strain control mode at a loading speed of 0.5 mm/min.

## 3. Test results and analysis

### 3.1 Test results

Tests were performed according to the experimental design. The uniaxial compressive strengths (UCSs) of the specimens aged for 3, 7, and 28 d were measured to determine the influences of the three factors on the strength of backfill materials in coal mines and their optimal composition. The obtained results are listed in Table 6.

**Table 6:** Results of the orthogonal tests performed in this study.

Sample	NaOH content/%	Water solid ratio	Fibre content/‰	UCS/MPa		
				3 d	7 d	28 d
FRGCT-1	1	0.24	4	0.61	1.66	3.58
FRGCT-2	1	0.28	5	0.82	2.43	4.36
FRGCT-3	1	0.32	6	0.66	1.811	3.88
FRGCT-4	2	0.24	5	1.12	3.18	4.59
FRGCT-5	2	0.28	6	1.32	3.73	5.11
FRGCT-6	2	0.32	4	1.19	3.04	4.76
FRGCT-7	3	0.24	6	1.59	3.61	5.29
FRGCT-8	3	0.28	4	2.36	4.31	6.04
FRGCT-9	3	0.32	5	2.11	4.08	5.77

### 3.2 Polarisation and ANOVA results

Based on the obtained results, the extreme deviation and variance of the mean value of the specimen UCS at the level of each factor were calculated. The influence of each factor on the test results was determined from the extreme deviation, and the significance of each factor was determined from the variance.

The orthogonal test results showing the effects of different factors on the 3-d UCS of the fibre-reinforced geopolymer-cemented tailing (FRGCT) specimens are presented in Table 7.

**Table 7:** Orthogonal test results for the 3-d UCS of the investigated samples.

Element	NaOH content/%	Water—solid ratio	Fibre content/‰
K1	1.027	1.343	1.420
K2	1.330	1.658	1.593
K3	2.120	1.477	1.463
Range	1.093	0.314	0.173
Primary and secondary orders	sodium hydroxide content > water-solid ratio > fibre content		
Superior level	A3	B2	C2
Optimal combination	A_3_B_2_C_2_		
Square of deviance	1.911	0.148	0.049
Degree of freedom	2	2	2
F-ratio	3.601	0.279	0.092
F_0.10_ critical value	3.110		
Significance	(*)	(–)	(–)

The orthogonal test results obtained for the 7-d UCS of the FRGCT samples are listed in Table 8.

**Table 8:** Orthogonal test results for the 7-d UCS of the investigated samples.

Element	Sodium hydroxide content/%	Water—solid ratio	Fibre content/‰
K1	1.967	2.817	3.003
K2	3.317	3.490	3.230
K3	4.000	2.977	3.050
Range	2.033	0.673	0.227
Primary and secondary orders	sodium hydroxide content > water—solid ratio > fibre content		
Superior level	A3	B2	C2
Optimal combination	A_3_B_2_C_2_		
Square of deviance	6.424	0.742	0.086
Degree of freedom	2	2	2
F-ratio	3.531	0.408	0.047
F_0.10_ critical value	3.110		
Significance	(*)	(–)	(–)

The orthogonal test results obtained for the 28-d UCS of the FRGCT samples are listed in Table 9.

**Table 9:** Orthogonal test results for the 28-d UCS of the investigated samples.

Element	Sodium hydroxide content/%	Water—solid ratio	Fibre content/‰
K1	3.940	4.487	4.793
K2	4.820	5.170	4.907
K3	5.700	4.803	4.760
Range	1.760	0.683	0.147
Primary and secondary order	sodium hydroxide content > water—solid ratio > fibre content		
Superior level	A3	B2	C2
Optimal combination	A_3_B_2_C_2_		
Square of deviance	4.646	0.702	0.035
Degree of freedom	2	2	2
F-ratio	3.451	0.521	0.026
F_0.10_ critical value	3.110		
Significance	(*)	(–)	(–)

**Fig 8:**
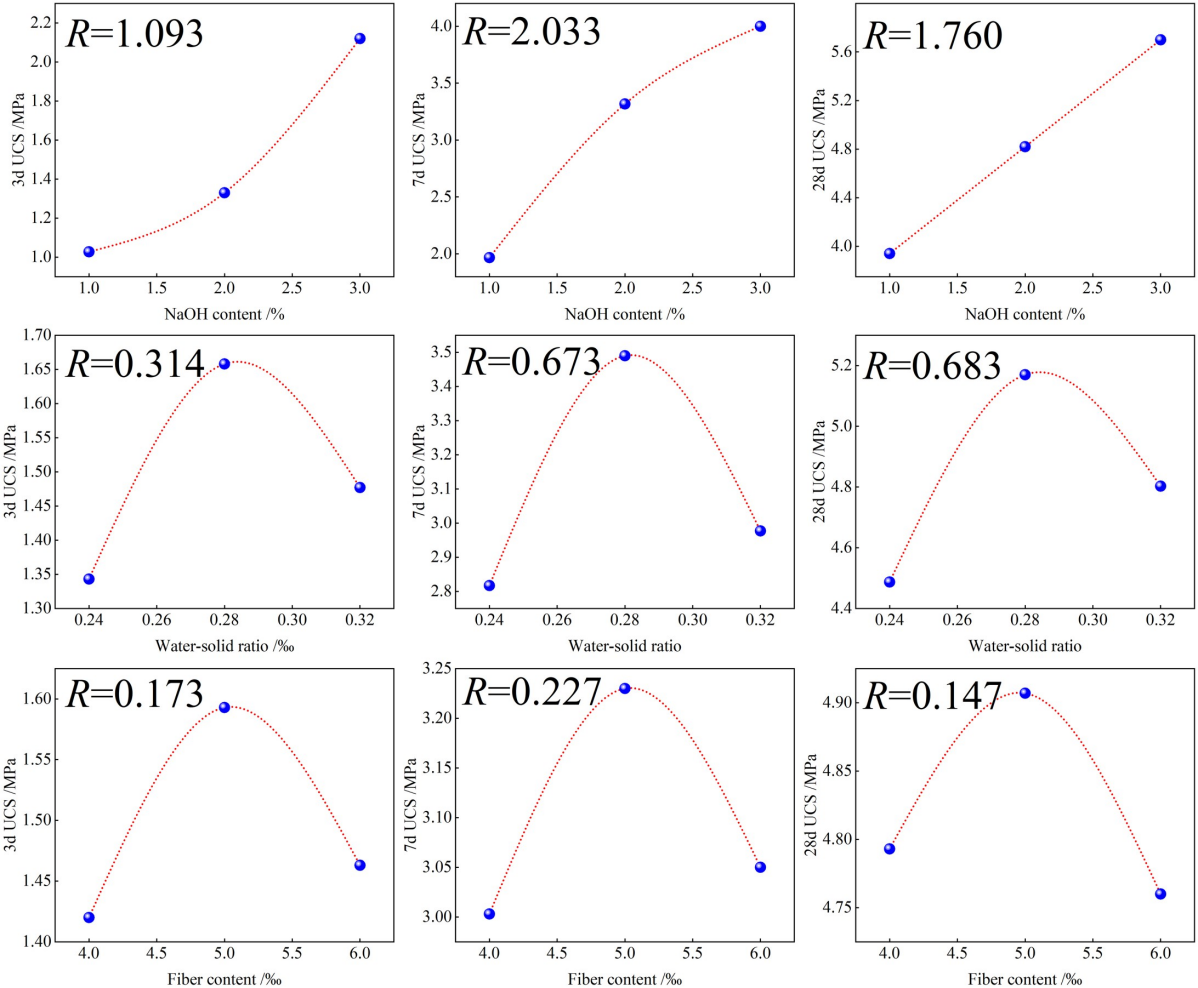
Influences of various factors on the average UCS.

The results of the test range and variance analysis suggest that the primary and secondary degrees of influence on the sample UCS after 3, 7, and 28 d of aging are the sodium hydroxide content, water—solid ratio, and fibre content. To examine the degree of influence of each factor in more detail, the level of each factor was plotted along the x-axis, and the average UCS was plotted along the y-axis (Fig 8) to determine compressive failure strengths. The following equation was used to calculate range *R*:

$$R=\text{max}\left(\bar{R_{c}}\right)-\text{min}\left(\bar{R_{c}}\right)$$ (1)

where *R* is the range, and *Rc* is the mean value of the UCS.

Fig 8 shows that with an increase in NaOH content, the UCS exhibits a gradually increasing trend. At a NaOH content of 3%, the UCS of the sample aged for 28 d reaches a local maximum. This occurs because at a low alkali amount, the driving force of the polymerisation reaction is low, and the degree of polymerisation of the generated product is low, resulting in an insufficiently dense material structure after hardening. With an increase in alkali content, the pH of the polymerisation reaction increases. A higher solution pH promotes the dissolution of Ca, Si, Al, and other elements, causing the dissolution of a larger amount of grey bodies, which produces silicate and aluminate monomers, accelerating the polymerisation reaction, generating a larger gel amount, and increasing the UCS. As shown in Fig 8, the UCS of the studied samples first increases and then decreases with an increase in the water—binder ratio. An appropriate water—binder ratio can increase the activity of the slag and fly ash and promote the polymerisation reaction; hence, the UCS of the geopolymer reaches a maximum. However, when the water—binder ratio is substantially high, although the fly ash and slag cementitious materials can be fully polymerised, the water content in the alkali solution is extremely high, resulting in a decrease in the pH of the alkali solution, which is detrimental to the fly ash and slag activity. The dissolution rate of the elements in the raw materials decreases, which reduces the reaction rate of the system and reaction degree. Meanwhile, the amount of the hydration products formed and structural compactness decrease, which ultimately decreases the specimen strength. However, the amount of free water in the solid waste matrix paste system gradually increases, and the excess free water remains inside the system. When water evaporates, voids are generated inside the sample structure; consequently, internal defects are created, and the geopolymer compactness and UCS are reduced. These voids lead to an uneven stress distribution when the sample is subjected to an external force, and the stress is concentrated near the voids, which quickly destroys the sample. Fig 8 also shows that with an increase in the fibre content, the UCS first increases and then decreases. At a low fibre content, the fibres inside the matrix are discrete, and their effect is weak. After increasing the fibre content, the fibres form a three-dimensional staggered structure, which helps absorb energy under loading. However, when the fibre content is too high, fibres easily tangle into groups inside the specimen; as, these do not contribute to the specimen tensile properties the UCS decreases.

The optimal level combination determined from the orthogonal test range and variance analysis results is A3B2C2, which corresponds to a NaOH content of 3%, water—solid ratio of 0.28, and fibre content of 5‰.

### 3.3 FRGCT failure process

**Fig 9:**
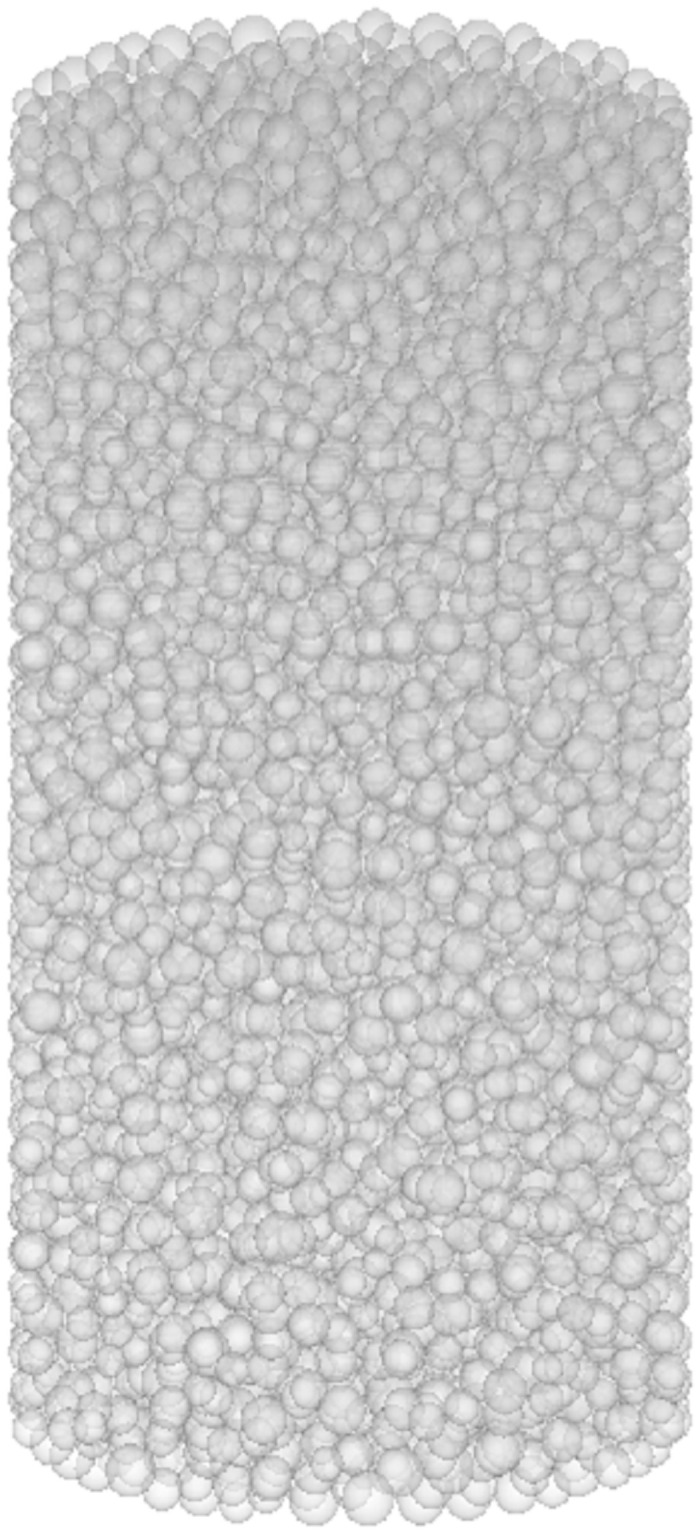
Initial state.

**Fig 10:**
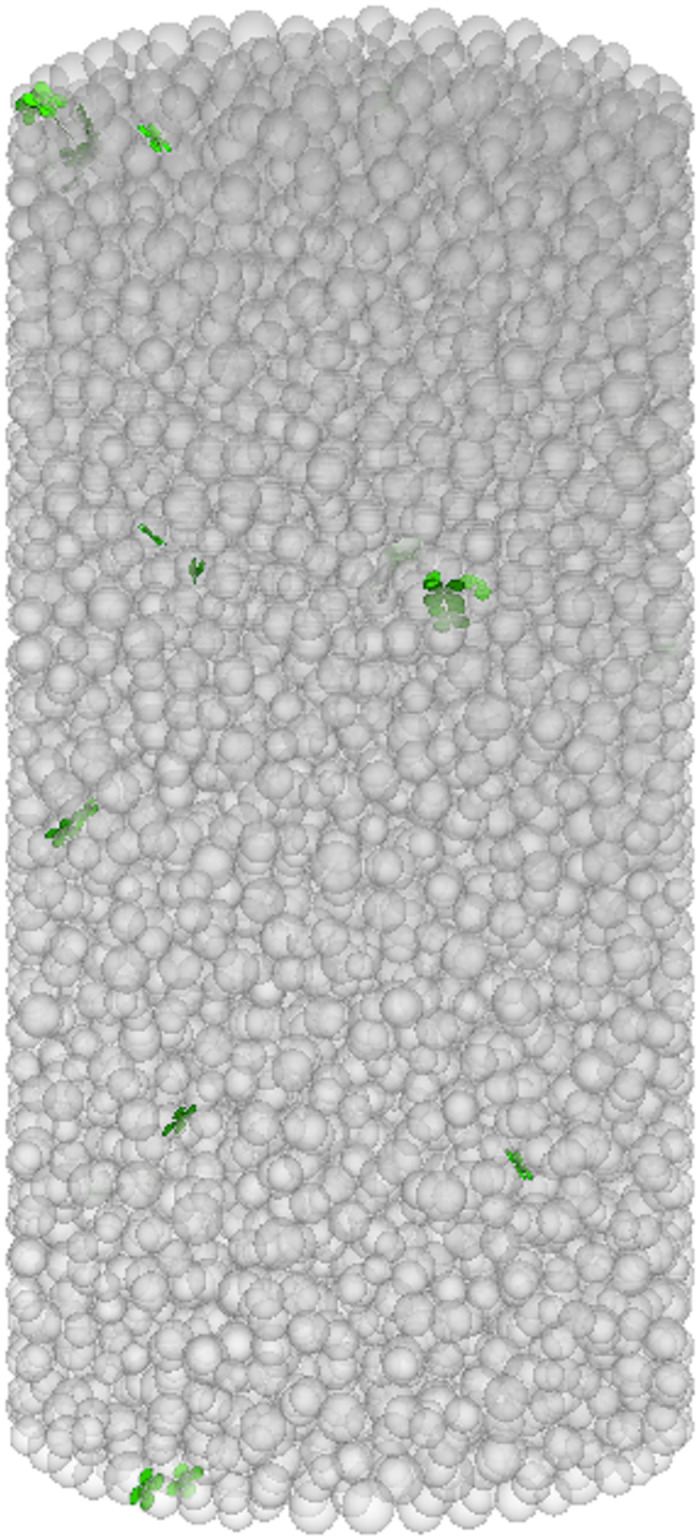
Fissure development.

**Fig 11:**
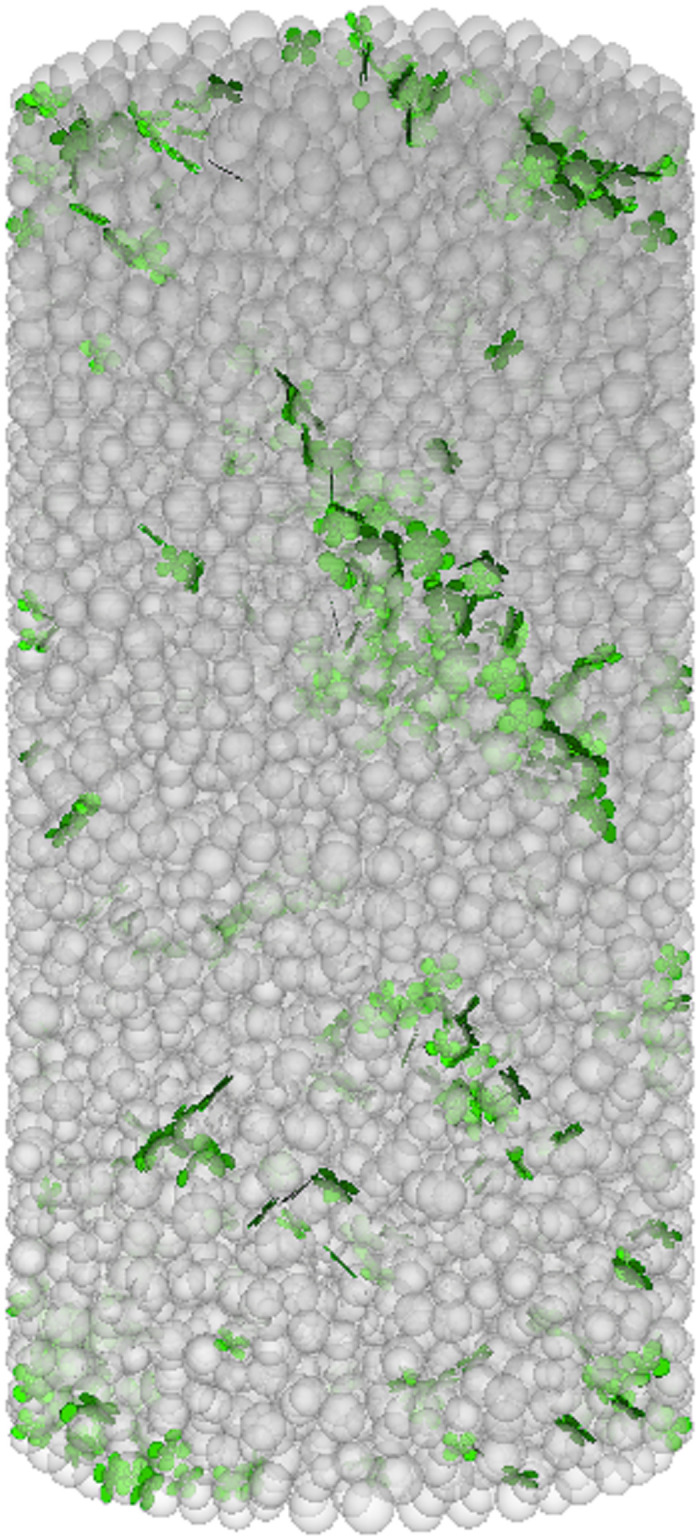
Crack propagation.

**Fig 12:**
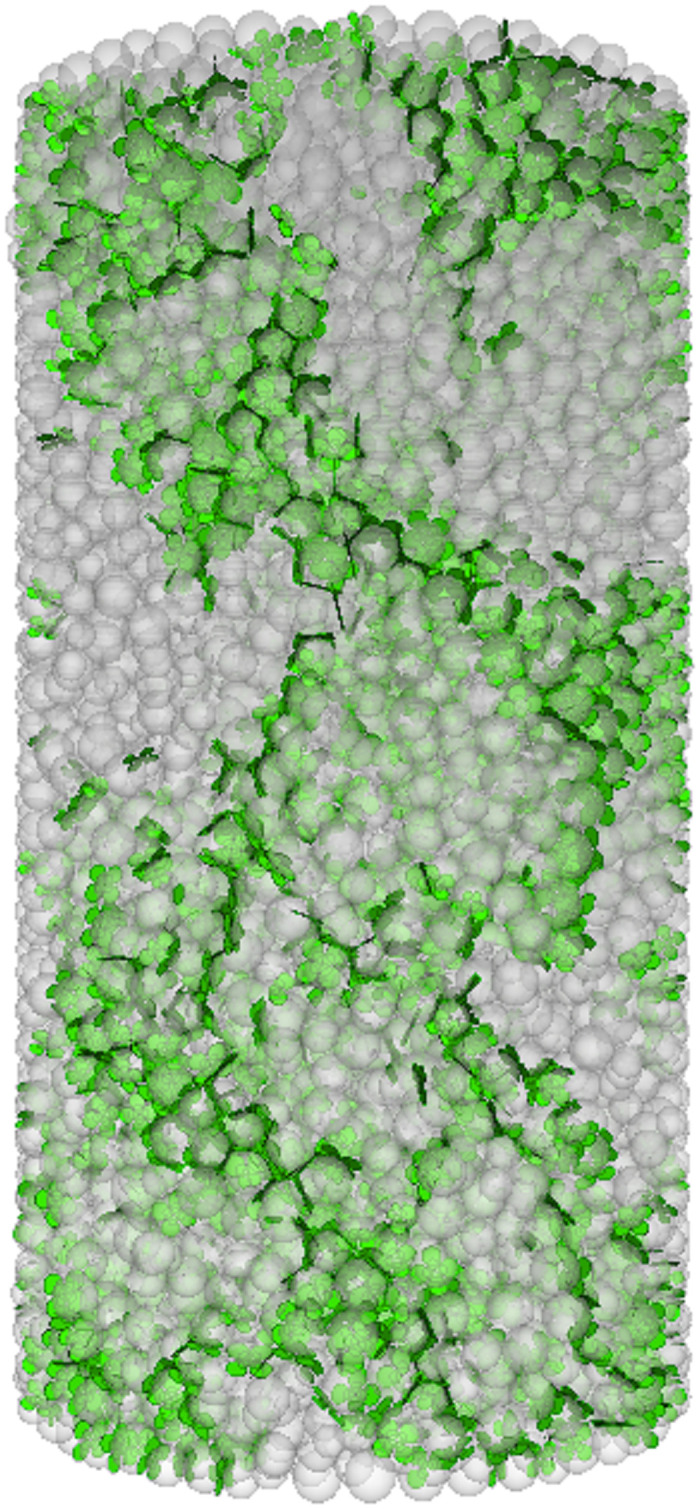
Fracture penetration.

The numerical simulation of the failure mode evolution process of FRGCT specimens is shown in Figs 9–12. During the loading process of the specimens, cracks developed in three stages. Initially, the loading was in the linear-elastic stage, followed by the yield stage. Small cracks appeared on the surface of the FRGCT specimen. As the deformation continued to increase, the cracks in the FRGCT specimen gradually increase and became more connected, with the overall failure mode presenting shear failure.

Under loading, the particles inside the FRGCT specimen are displaced in different directions, and numerous shear cracks form in the specimen body. For the well-cemented polymer specimens doped with fibres, damage occurs only under large loads when particles inside the polymer are sufficiently compacted, and apparent shear slip surfaces are present in the polymer structure. At a low fibre content, the specimen tends to undergo crushing damage, whereas at a high fibre content, the specimen tends to undergo shear damage. The displacement vector field distribution based on a discrete element model reflects the stable mechanical response of polypropylene fibres. When the fibre content is low, the bond strength between the particles is low, and internal particle dislocation and slip are prone to occur. Thus, the failure probability is high. With the increase in fibre content, the cracks inside the sample during failure become finer, indicating that the incorporation of polypropylene fibres into the backfill can improve its anti-cracking ability, effectively increasing the energy absorption capacity during deformation and specimen deformation resistance; thus, the geopolymer–fibre backfill material exhibits high toughness and UCS.

### 3.4 Stress–strain curve analysis

**Fig 13:**
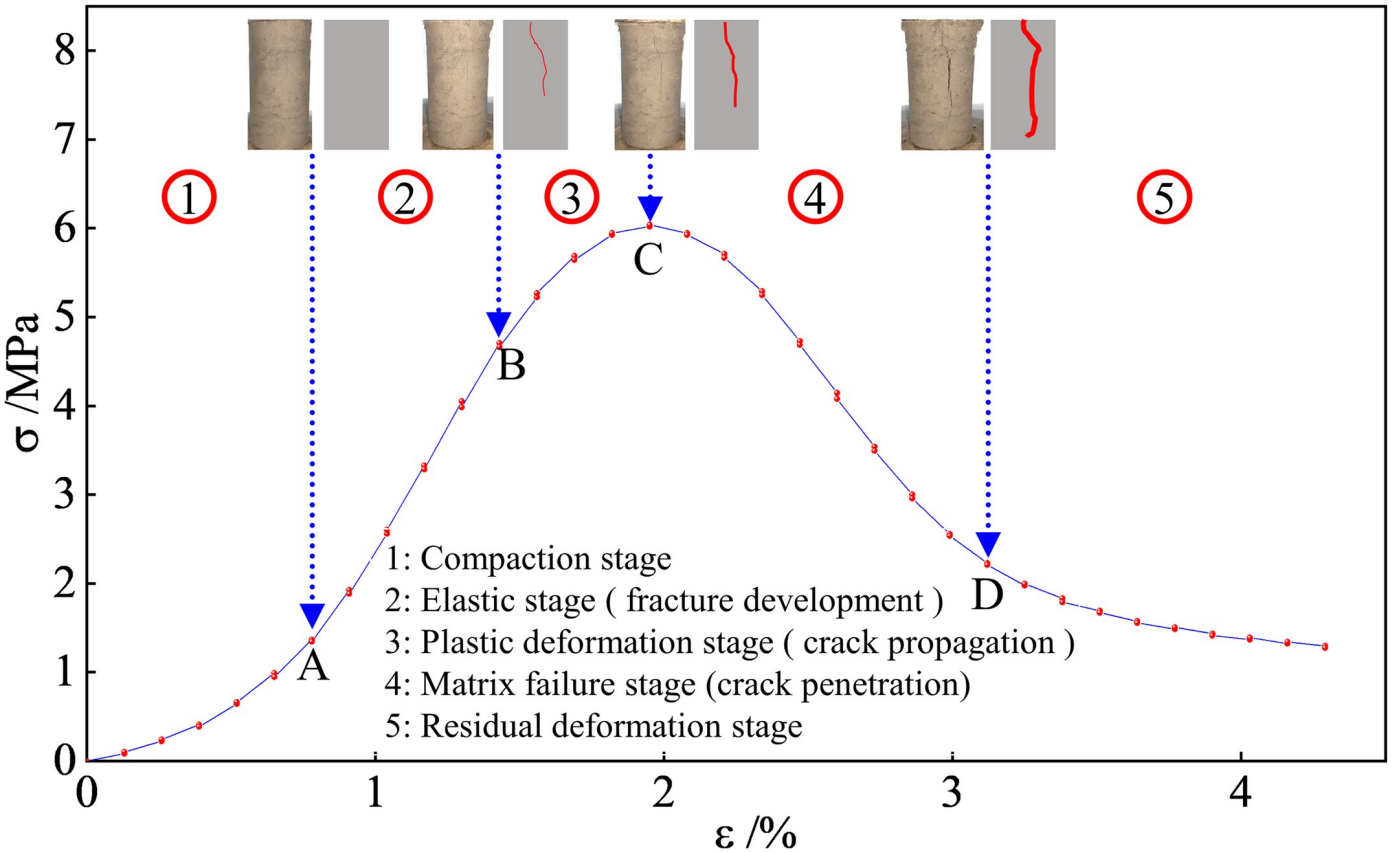
Stress–strain curve of the FRGCT-8 specimen.

The stress–strain curve of the FRGCT-8 specimen recorded during loading is depicted in Fig 13.

Uniaxial compressive stress–strain curves are frequently used in force analyses and theoretical calculations. To simplify the analysis procedure, dimensionless coordinates, i.e. *x = ε/ε*_*p*_, *y* = *σ*/*σ*_*p*_, were employed, where *ε*_*p*_ is the average peak strain, *ε* is the sample strain, *ε* is the average peak stress, and *σ*_*p*_ is the sample stress.

The equation for the rising segment of the FRGCT stress–strain curve is [22] as follows:

$$y=ax+bx^{2}+cx^{3}\;(0\leq x\leq 1)$$ (2)

The equation for the descending section of the stress–strain curve of the FRGCT sample can be written as follows:

$$y=\frac{x}{d{\left(x-1\right)}^{2}+x}$$ (3)

The slope of curve is the elastic modulus of materials:

$$a={\left.\frac{dy}{dx}\right|}_{x=0}=\frac{d\sigma /d\sigma_{p}}{d\varepsilon /d\varepsilon_{p}}={\left.\frac{d\sigma }{d\varepsilon }\right|}_{\varepsilon =0}\frac{d\varepsilon_{p}}{d\sigma_{p}}=\frac{E_{c}}{E_{p}}$$ (4)

At *x* = 1, *y* = 0.98

a+b+c=0.98 (5)

At *x* = 1 and *dy/dx* = 0, the derivative of (2) is

a+2b+3c=0 (6)

According to (4)–(6),

$$a=\frac{E_{C}}{E_{0}},b=2.94-2a,c=a-1.96$$ (7)

By substituting a, b, and c into (2), the equation for the rising section of curve of the FRGCT sample can be written as follows:

$$y=ax+\left(2.94-2a\right)x^{2}+\left(a-1.96\right)x^{3}$$ (8)

**Fig 14:**
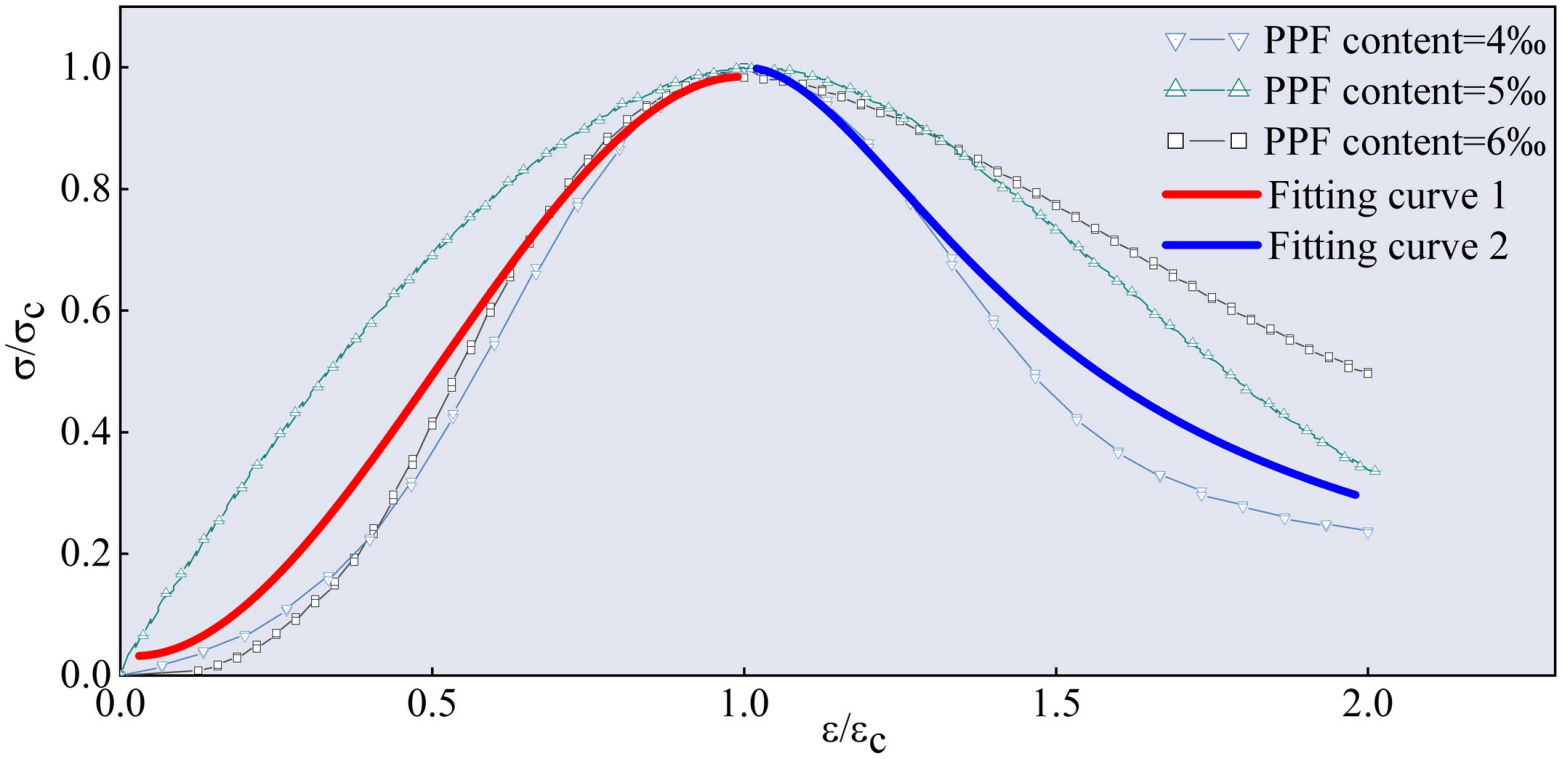
Average stress–average strain and theoretical curves of various samples.

Eqs (2) and (3) were used to fit the complete stress–strain curves of the FRGCT specimens under uniaxial compression. The fitting curve, experimental curve, a value, d value, and fitting correlation are shown in Fig 14 (owing to the limited space, only the fitting data for the FRGCT-7, FRGCT-8, and FRGCT-9 specimens are provided in this paper). The fitting procedureindicates that the mass content range of polypropylene fibres was 4 ≤ Vf ≤ 6%, *a* = −0.144, and *d* = 4.881. Thus, the equation for the rising section of the curve of the FRGCT sample is as follows:

$$y=-2.108x^{3}+3.232x^{2}-0.144x$$ (9)

The equation for the descending section of t curve of the FRGCT sample is as follows:

$$y=\frac{x}{4.881{\left(x-1\right)}^{2}+x}$$ (10)

## 4. Microscopic characterisation of backfill materials

**Fig 15:**
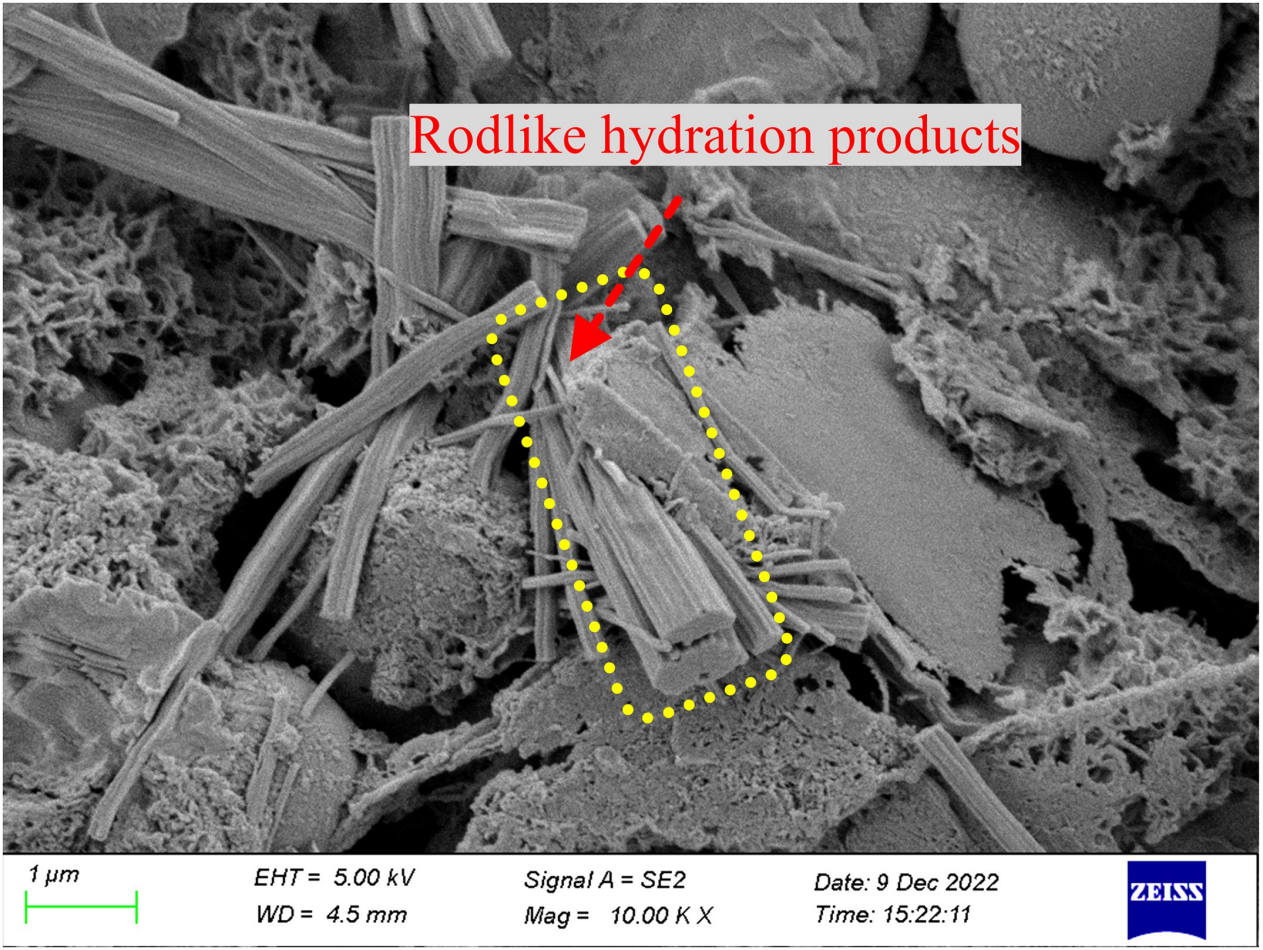
SEM images of 3d geopolymer samples.

**Fig 16:**
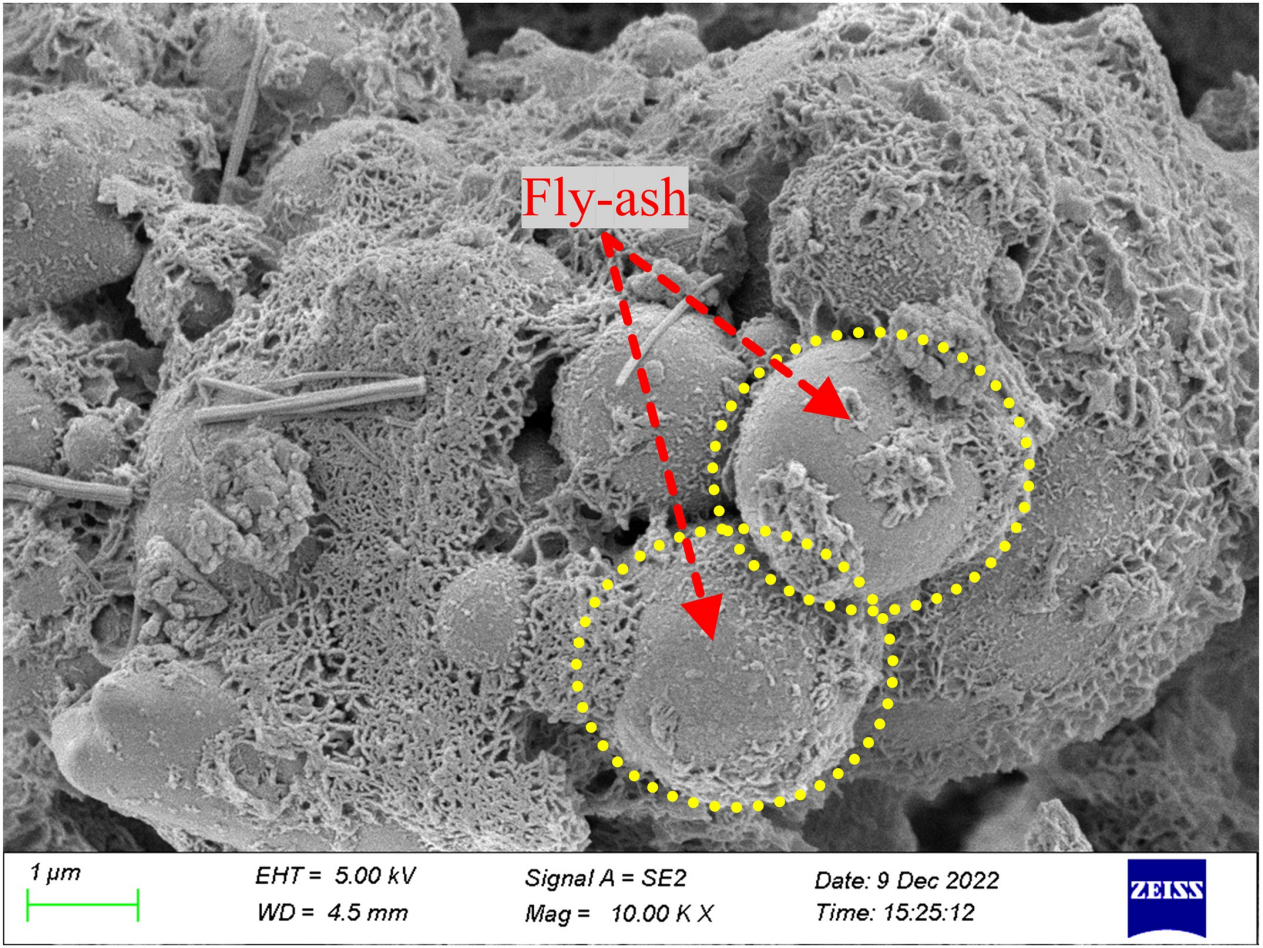
SEM images of 7d geopolymer samples.

**Fig 17:**
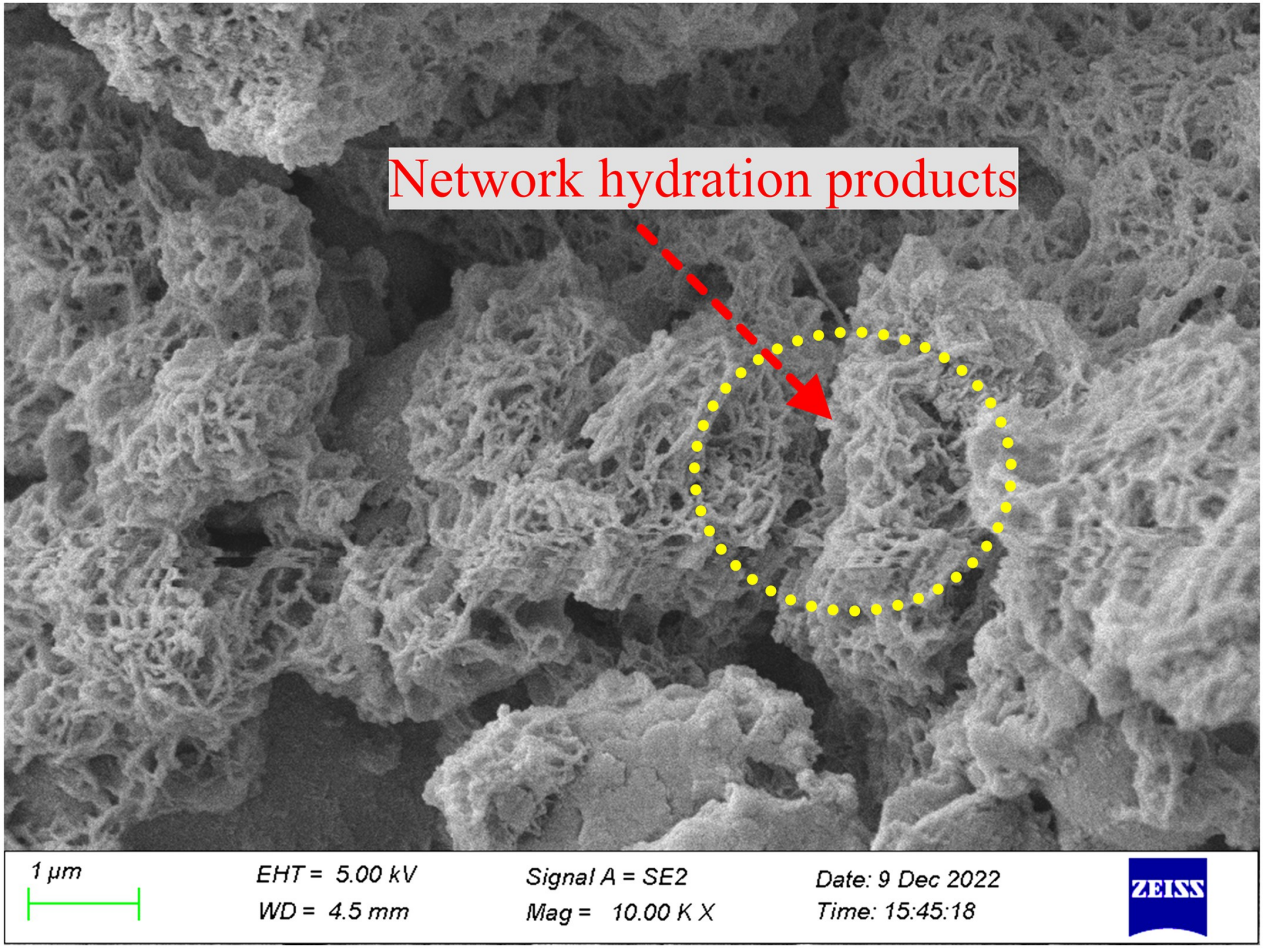
SEM images of 28d geopolymer samples.

As shown in Figs 15–17, observations using electron microscopy reveal that the overall structure of the hydration products is considerably dense. Compared to the 3-d hydration products, the hydration products at 28-d have a more compact overall structure, presenting an amorphous form, and are covered with a large amount of hydration gel. Regardless of curing age, the hydration products contain fly ash particles. In the 3-d hydrated paste, most fly ash particles have relatively smooth surfaces and form many rod-like hydration products, with a weaker bond between the hydration products and the particles. However, in the 28-d hydration products, the surface of the fly ash particles is covered with a large amount of hydration products, and these particles are tightly wrapped by the hydration products.

The fly ash was rich in aluminium, forming hydrated aluminosilicates with a shelf structure, which primarily contributed to the later strength of the backfill material. In contrast, the slag functioned as a calcium source, leading to the formation of a C–S–H gel, which was necessary for the early strength of the backfill material. Furthermore, the flowability of the mixture was enhanced by the flat surface of the fly ash particles. Consequently, the combination of these two materials exhibits high strength and workability.

The microstructures of the FRGCT specimens with different doping ratios after destruction were observed using SEM. Local magnification was performed by changing from a low-magnification mode to a high-magnification mode when a typical region was investigated, and sample photos were recorded. The SEM images used in this investigation have magnifications ranging from 100× to 1000×.

**Fig 18:**
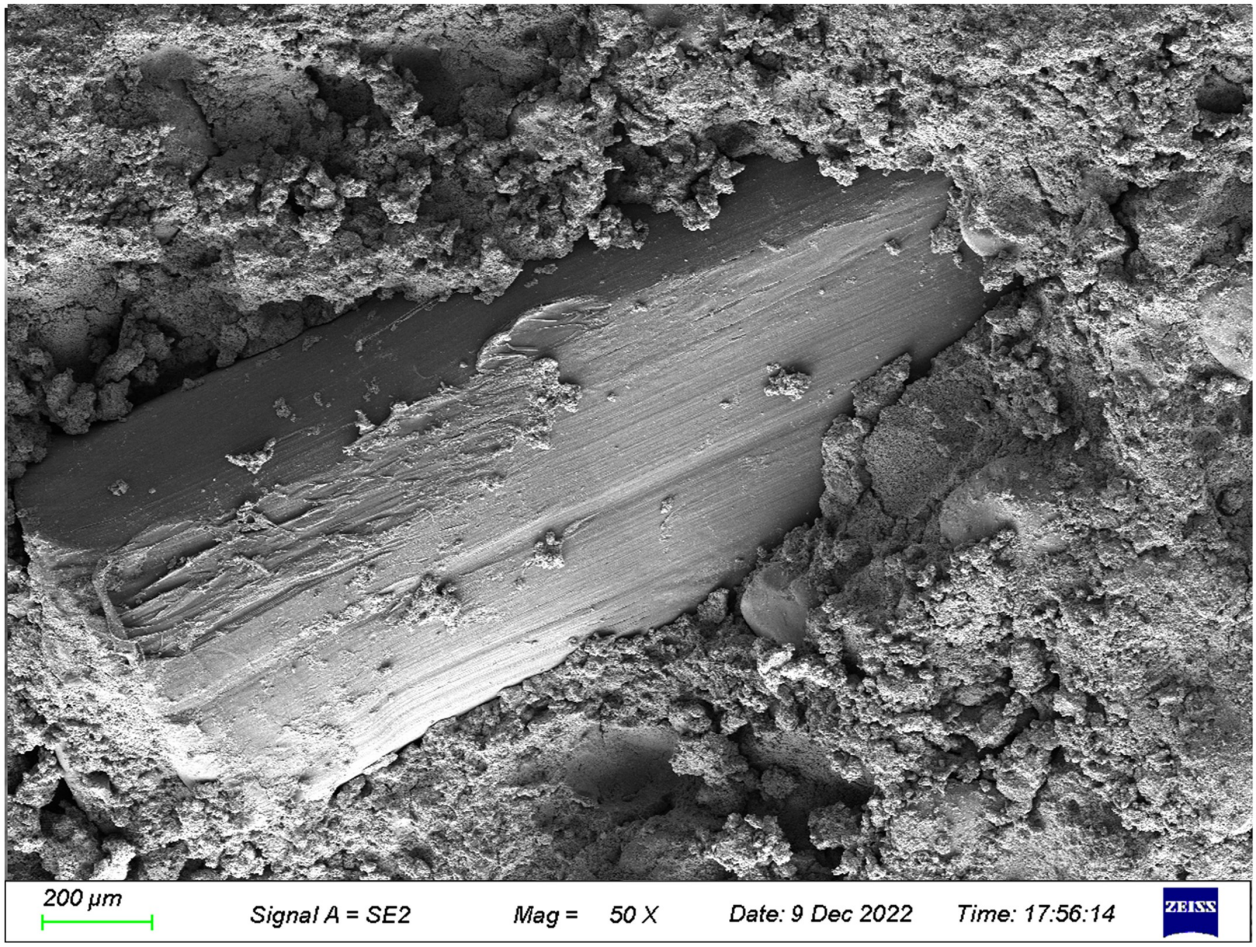
SEM images of the FRGCT specimens with fibre content of 4‰.

The SEM image of the ground polymer with 4‰ fibres is presented in Fig 18. The figure shows that the fibres embedded into the matrix of the cementitious material, randomly distributed, and highly discrete. During the initial phase of the FRGCT specimen aging process, hydrides continue to form and fill the spaces between the fly ash particles and between fibres and fly ash particles, enhancing their interfacial cohesiveness. Fibres and tailings sand particles are mixed by extrusion and wrapping. Isolated fibres of low fibre content are buried with increasing grip area when they are firmly enveloped in fly ash particles. The fibres are in the tensile condition when the specimen is deformed or damaged by an external load, and the relative displacement of the particles is reduced owing to the bonding and friction forces that limit their relative sliding movements.

**Fig 19:**
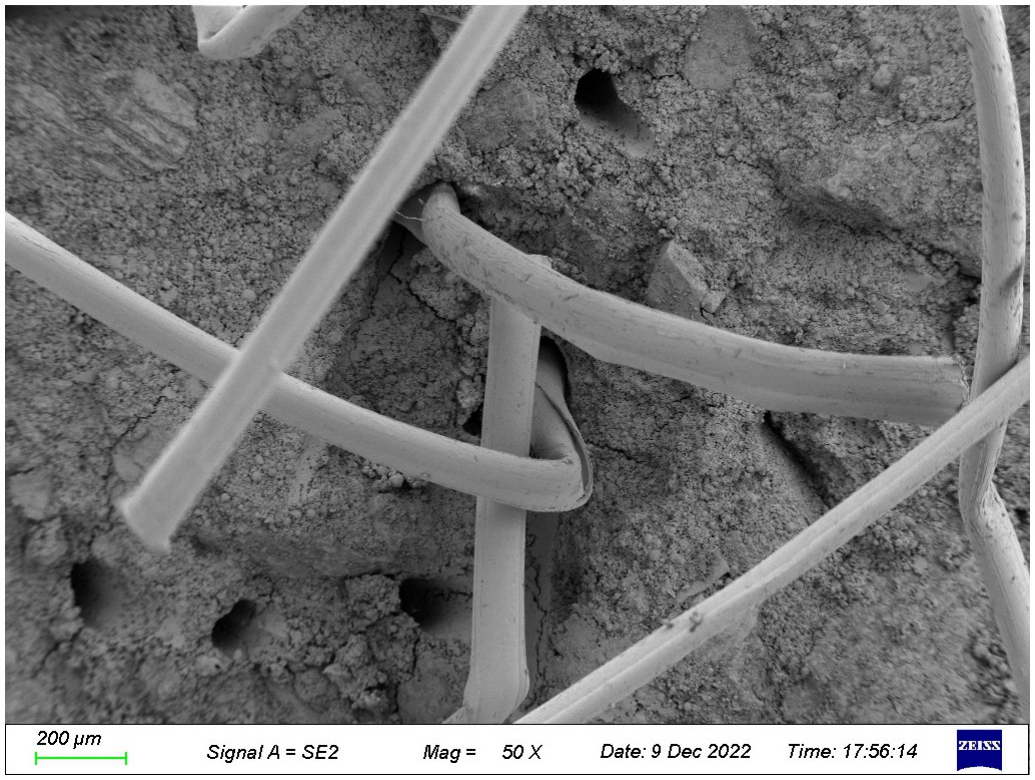
SEM images of the FRGCT specimens with fibre content of 5‰.

The SEM image of the specimen with a fibre content of 5‰ is shown in Fig 19. Fibres in the polymer matrix exhibit a uniformly chaotic crisscross distribution, which is similar to that of the roots embedded in soil, to form a more stable three-dimensional spatial structure. When fly ash sand particles are under the action of an external load causing the misalignment and rearrangement of the fibre network, they disperse the load to other areas of the specimen more effectively, reducing stress concentration by bearing stress, thereby improving the specimen stability. The hydration reaction between fly ash particles, tailings sand particles, and fibres forms crystalline products and crystals adhering to the fibre surface (Fig 19). The hydration products blend to form a porous dense mesh structure that strengthens the interfacial contacts between the fly ash particles and the fibres as well as their adherence. Simultaneously, discrete fibres are cemented into a mesh structure and a zone of anchoring is formed around them by the fibre-wrapped hydrated crystals, which resemble hard shell layers in shape. This process increases the stiffness of the fibres, which increases the adhesion and stabilization of the fibres and fly ash particles in the non-homogeneous material. Consequently, the toughness and integrity of the geopolymer—fibre backfill material are increased because the formation and development of cracks are effectively suppressed and the fibres and fly ash particles do not easily slide relative to each other under loading.

**Fig 20:**
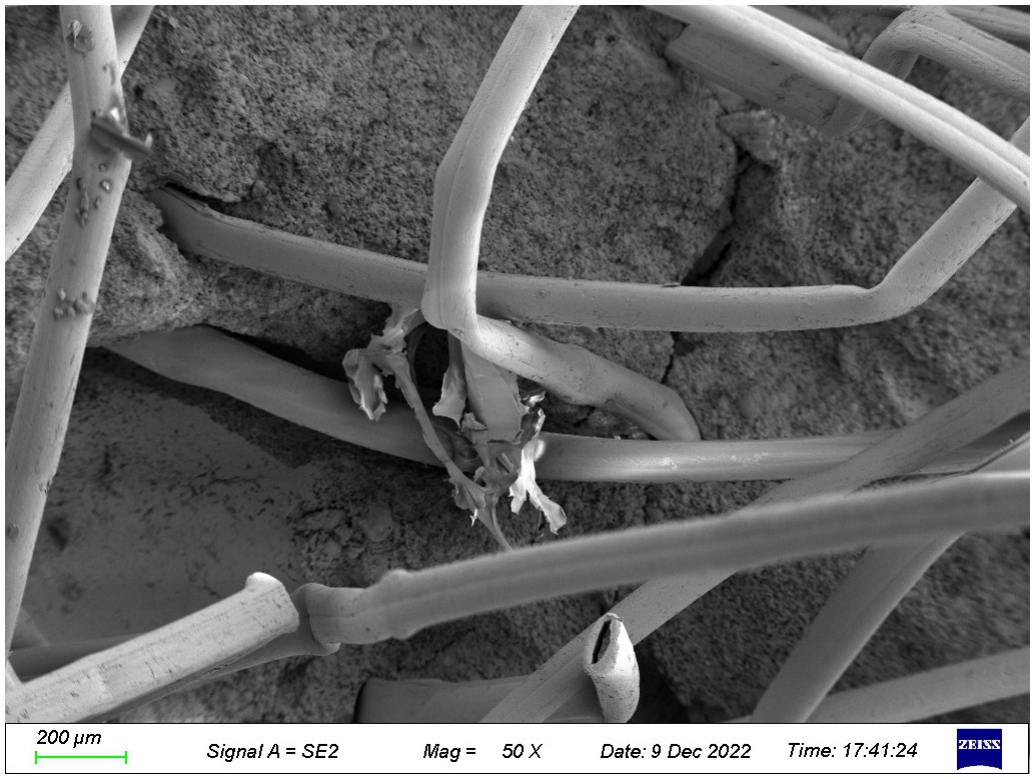
SEM images of the FRGCT specimens with fibre content of 6‰.

Fibres are less evenly distributed and have a lower degree of dispersion in the sample when the fibre content reaches 6% (Fig 20); the degree of cementing hydration between particles is greatly decreased. In Fig 20, some of the fibres are orientedparallel to one another, and the fibres are partially out of contact with the polymerised products, which reduces the gripping area. The gelling material matrix be-comes looser and the overall performance of the material deteriorates because the water-containing compounds are unable to fill the spaces between the particles and fibres during the hydration reaction. The overall structure of the sample is rapidly destroyed when subjected to an axial load because the overlaid weak layer’s fibres slip and break, with a minimal reinforcing effect.

## 5. Conclusions

This study examined the effects of various parameters on the UCS of fly ash composite cement samples that were generated through the homogenous mixing of polypropylene fibres with the cementing material. SEM was used to analyse the composite structure’s interfacial interactions at the microscopic level. The following conclusions were drawn based on the results:

1. The strength model established using the orthogonal design method exhibited a high accuracy and predictability. The effect of each factor on the backfill material was ranked as follows: sodium hydroxide content > water—solid ratio > fibre content. Moreover, a significant interaction was observed between these parameters, which necessitated the establishment of strict measurement accuracy requirements for the production and use of cementing materials. The optimal mix ratio with a 28-d UCS above 5 MPa was achieved at a sodium hydroxide content of 3%, water—solid ratio of 0.28, and fibre content of 5‰, which satisfies the requirements of deep backfill mining.
2. The displacement vector field distribution obtained via discrete element simulations using PFC3D software effectively reflected the real failure form of the studied FRGCT samples after a uniaxial compression test from a microscopic perspective. After adding polypropylene fibres, the peak stress—strain increased, and the maximum value was reached at a fibre content of 5‰, which increased the strength and ductility of the backfill before failure.
3. At a fibre concentration of 5‰, the blended system’s fibres were uniformly and randomly dispersed, displaying chaotic distribution features in three-dimensional space, according to SEM observations. Fibres met at several points, and the fibre-reinforced geopolymer matrix adopted a comparatively stable three-dimensional spatial structure. Nevertheless, at reaching a fibre content of 6%, the sample’s homogeneous fibre distribution rapidly diminished due to fibre agglomeration, thereby indirectly reducing the effective contact area between particles and fibres.

The results of this study can serve as a reference for similar studies related to mine management projects.

## Supporting information

S1 Data (ZIP)
