# Supplementary figures and images for: Mechanical properties of fibre-reinforced geopolymer-cemented tailings used as backfill
Source: PLoS One. 2024 Dec 5;19(12):e0314617. doi: 10.1371/journal.pone.0314617 (PMC11620621; doi:10.1371/journal.pone.0314617)

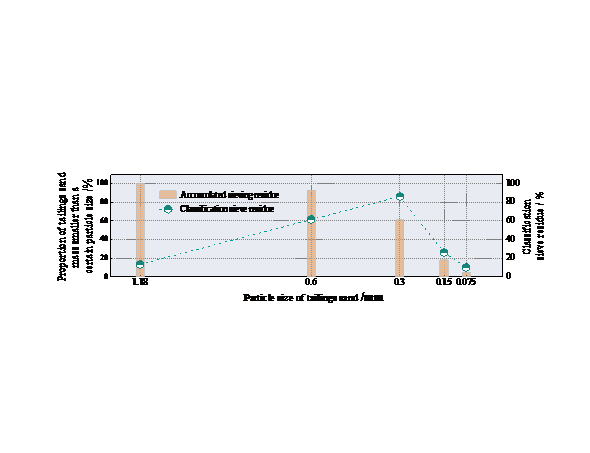

Supplement: S1 Data — (ZIP) [file pone.0314617.s001.zip › Date/Fig1.tif]

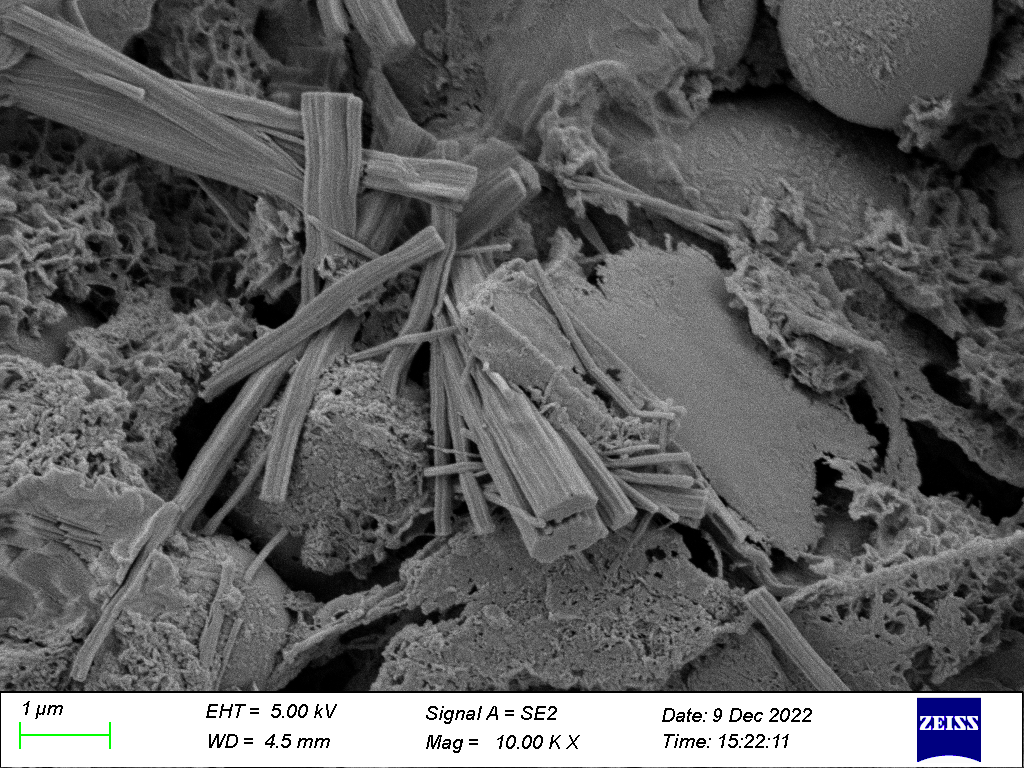

Supplement: S1 Data — (ZIP) [file pone.0314617.s001.zip › Date/Fig10-3d.tif]

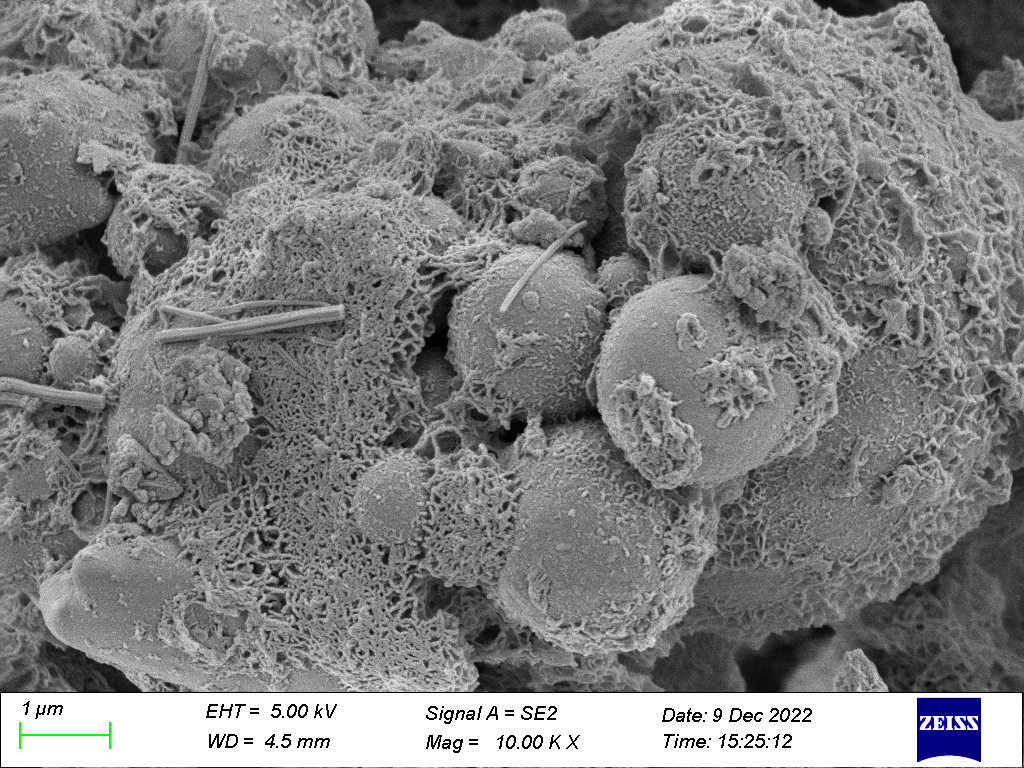

Supplement: S1 Data — (ZIP) [file pone.0314617.s001.zip › Date/Fig10-7d.tif]

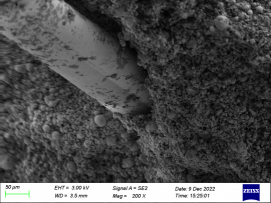

Supplement: S1 Data — (ZIP) [file pone.0314617.s001.zip › Date/Fig11-(a).tif]

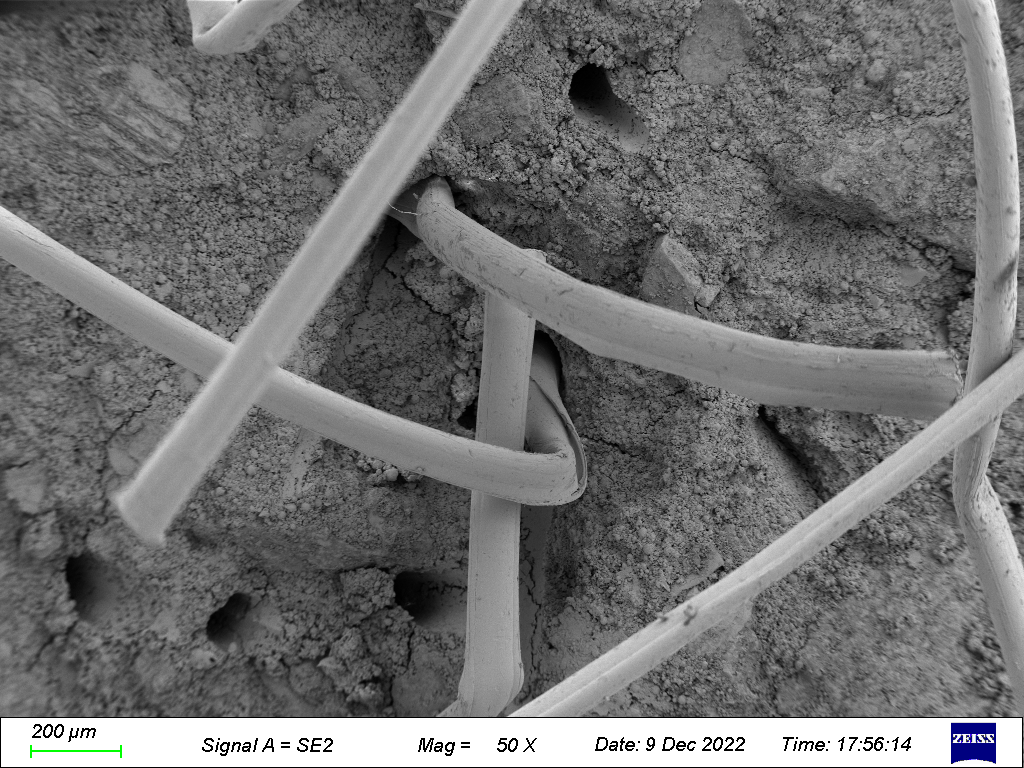

Supplement: S1 Data — (ZIP) [file pone.0314617.s001.zip › Date/Fig11-(b).tif]

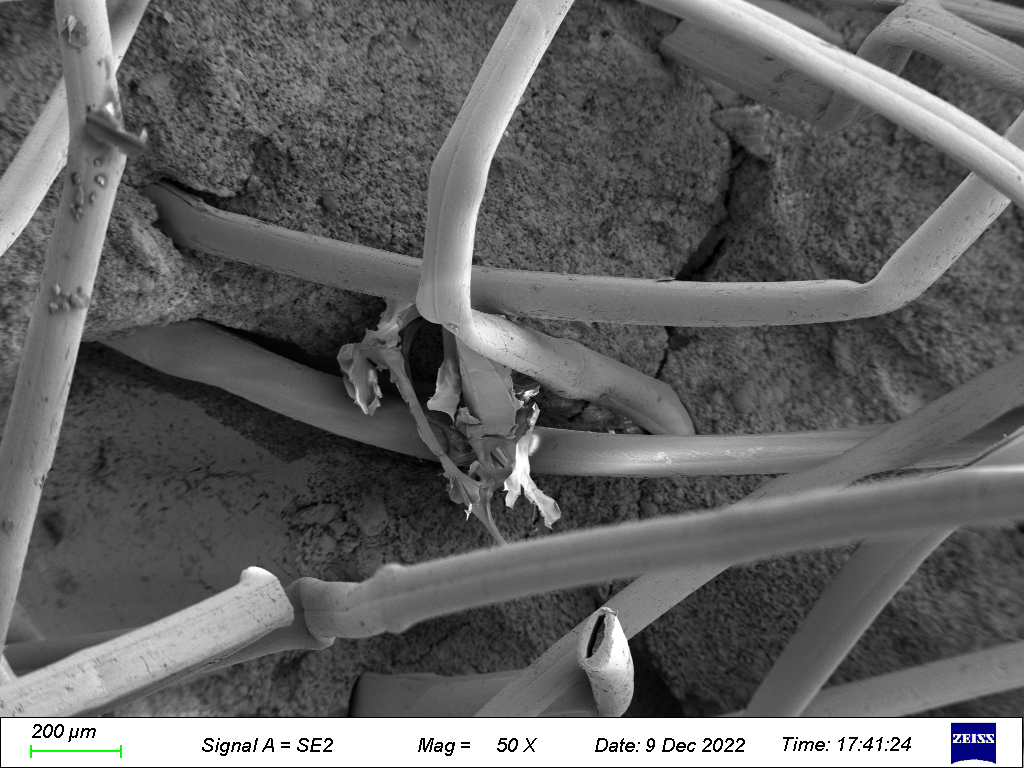

Supplement: S1 Data — (ZIP) [file pone.0314617.s001.zip › Date/Fig11-(c).tif]

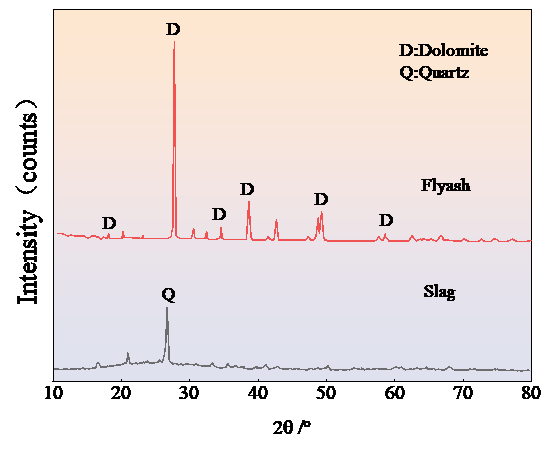

Supplement: S1 Data — (ZIP) [file pone.0314617.s001.zip › Date/Fig2.tif]

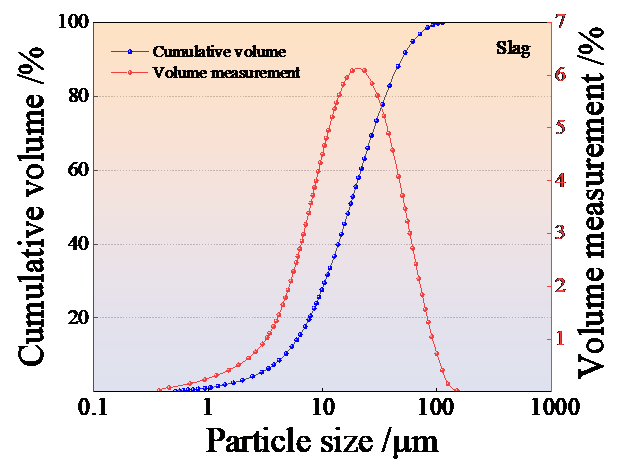

Supplement: S1 Data — (ZIP) [file pone.0314617.s001.zip › Date/Fig3-(a).tif]

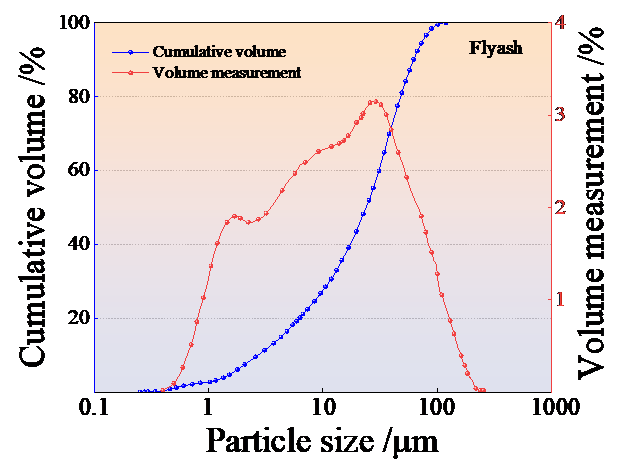

Supplement: S1 Data — (ZIP) [file pone.0314617.s001.zip › Date/Fig3-(b).tif]

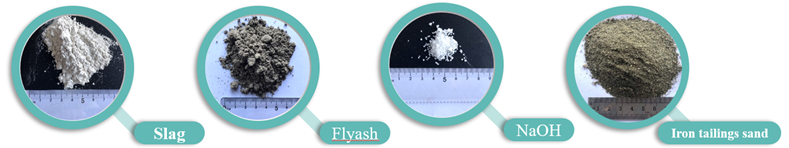

Supplement: S1 Data — (ZIP) [file pone.0314617.s001.zip › Date/Fig4.tif]

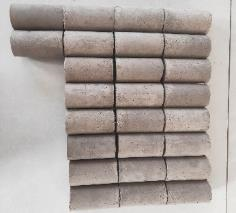

Supplement: S1 Data — (ZIP) [file pone.0314617.s001.zip › Date/Fig5-(a).tif]

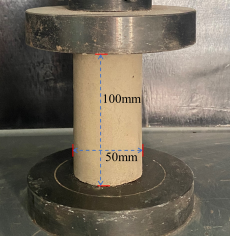

Supplement: S1 Data — (ZIP) [file pone.0314617.s001.zip › Date/Fig5-(b).tif]

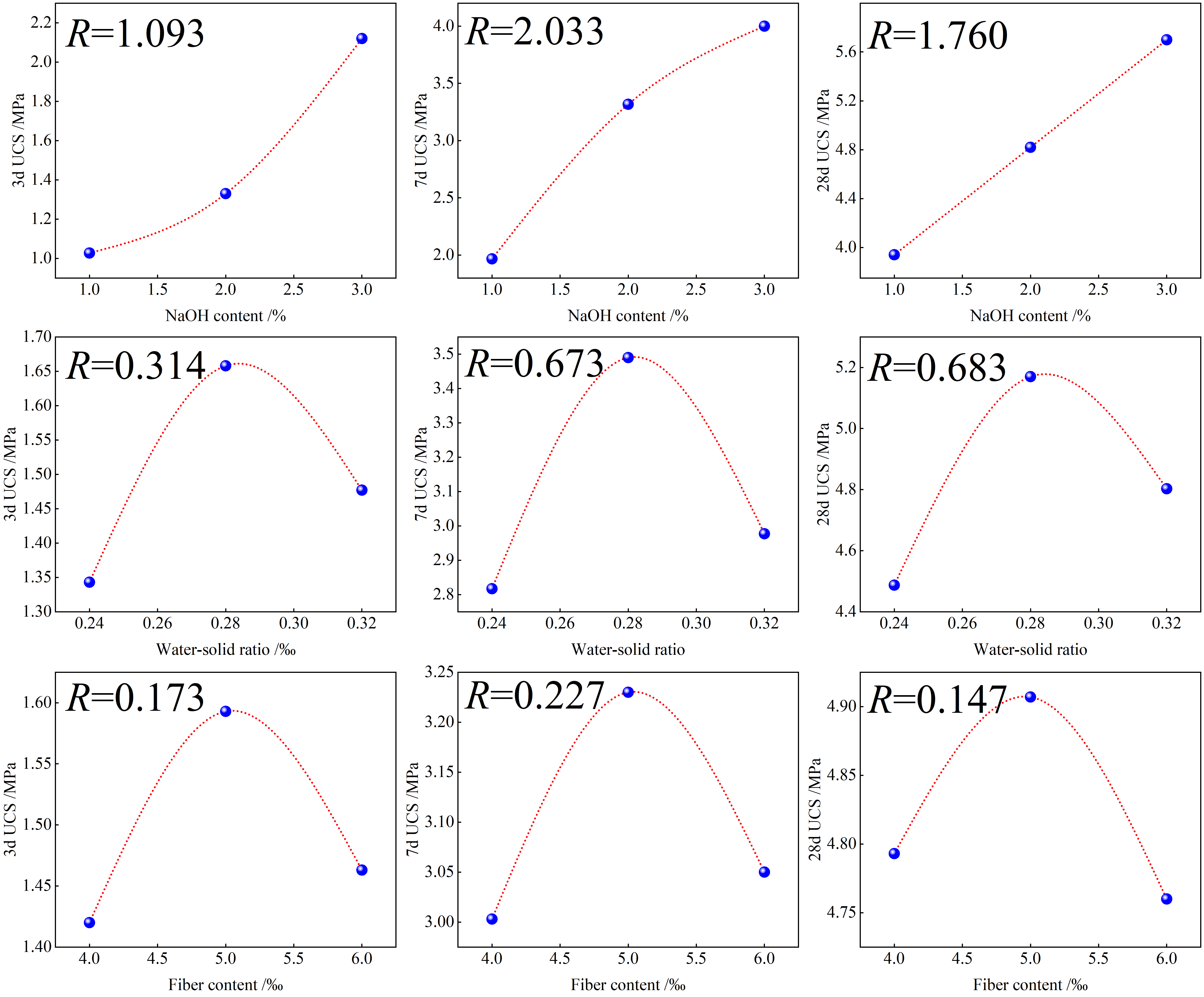

Supplement: S1 Data — (ZIP) [file pone.0314617.s001.zip › Date/Fig6.tif]

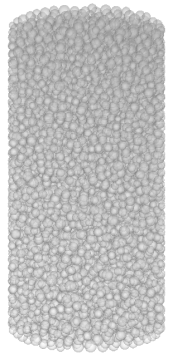

Supplement: S1 Data — (ZIP) [file pone.0314617.s001.zip › Date/Fig7-(a).tif]

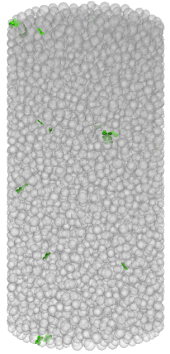

Supplement: S1 Data — (ZIP) [file pone.0314617.s001.zip › Date/Fig7-(b).tif]

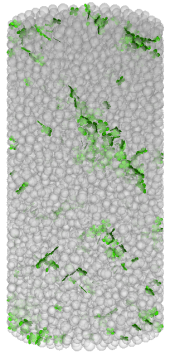

Supplement: S1 Data — (ZIP) [file pone.0314617.s001.zip › Date/Fig7-(c).tif]

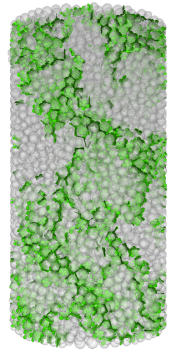

Supplement: S1 Data — (ZIP) [file pone.0314617.s001.zip › Date/Fig7-(d).tif]

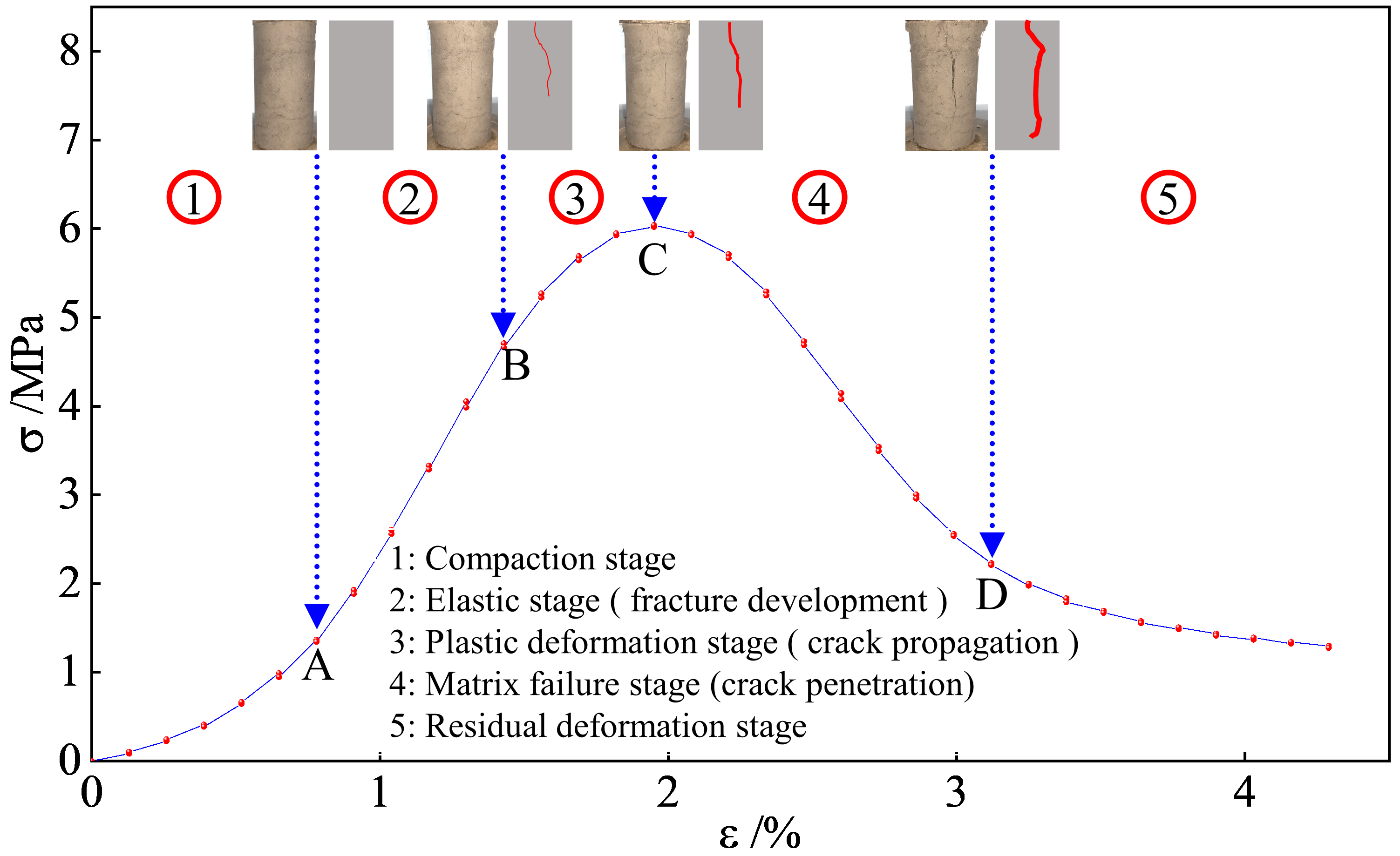

Supplement: S1 Data — (ZIP) [file pone.0314617.s001.zip › Date/Fig8.tif]

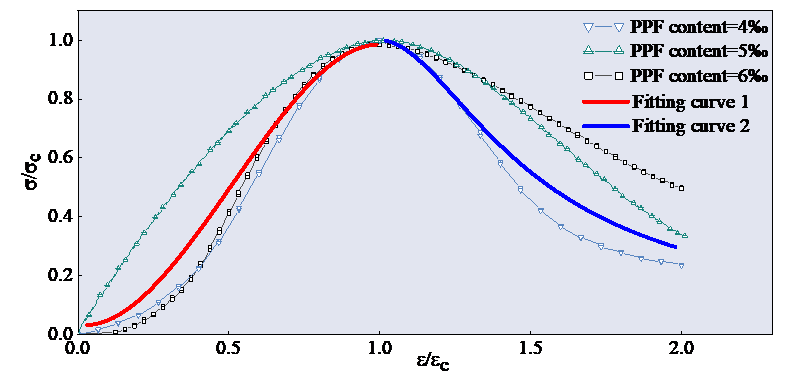

Supplement: S1 Data — (ZIP) [file pone.0314617.s001.zip › Date/Fig9.tif]
